# AKT Phosphorylation of FOXK2 at Ser398 Impacts Breast Cancer Cell Growth and Viability

**DOI:** 10.1096/fj.202600413RRR

**Published:** 2026-10-13

**Authors:** L. T. Carneiro, J. M. R. C. Nunes, G. De Gregoriis, B. S. Mendonça, W. M. Araújo, R. C. Maia, E. W. F. Lam, R. S. Carvalho, M. A. Carvalho, A. D. Sharrocks, G. Nestal de Moraes

**Affiliations:** ^1^ Laboratório de Sinalização e Biologia Tumoral, Instituto de Bioquímica Médica Leopoldo de Meis Universidade Federal do Rio de Janeiro (UFRJ) Rio de Janeiro RJ Brazil; ^2^ Divisão de Pesquisa Clínica Instituto Nacional de Câncer (INCA) Rio de Janeiro RJ Brazil; ^3^ Departamento de Biologia Animal–Instituto de Ciências Biológicas e da Saúde (ICBS)–Campus Seropédica Universidade Federal Rural do Rio de Janeiro (UFRRJ) Rio de Janeiro RJ Brazil; ^4^ Laboratório de Hemato‐Oncologia Celular e Molecular, Programa de Hemato‐Oncologia Molecular Instituto Nacional de Câncer (INCA) Rio de Janeiro RJ Brazil; ^5^ Department of Surgery and Cancer Imperial College London London UK; ^6^ Instituto de Ciências Biomédicas Universidade Federal do Rio de Janeiro (UFRJ) Rio de Janeiro RJ Brazil; ^7^ Laboratório de Genética Molecular Instituto Federal de Educação, Ciência e Tecnologia do Rio de Janeiro (IFRJ) Rio de Janeiro RJ Brazil; ^8^ School of Biological Sciences, Faculty of Biology, Medicine and Health University of Manchester Manchester UK

**Keywords:** AKT oncogenic kinase, breast cancer cell growth, drug resistance, FOXK2 phosphorylation, FOXK2 transcription factor

## Abstract

FOXK2 is a transcription factor known to mediate drug sensitivity in breast cancer. However, poor prognosis patients and drug‐resistant cells exhibit constitutively elevated levels of FOXK2 protein, which appears functionally inactive. Although emerging evidence suggests that FOXK2 is regulated by post‐translational modifications, the underlying mechanisms in breast cancer remain poorly understood. An *in silico* analysis identified AKT as a putative regulator of FOXK2, prompting the investigation of AKT‐mediated phosphorylation of FOXK2 in cancer. Our results show that elevated FOXK2 protein levels are associated with increased AKT phosphorylation and doxorubicin resistance. Modulation of AKT levels reduces FOXK2 at protein but not mRNA levels. Consistently, analysis of public breast tumor datasets revealed increased FOXK2 phosphorylation at different sites, including serine 398. Immunoblotting with an antibody against AKT substrates and lambda phosphatase treatment indicates that serine 398 in FOXK2 is an AKT phosphorylation site. Notably, the phosphodeficient FOXK2 mutant (S398A) impaired cell growth, metabolic cell viability, and clonogenic capacity, even in the presence of chemotherapeutic drugs. In summary, AKT regulates FOXK2 in drug‐resistant breast cancer cells, with serine 398 emerging as a novel phosphorylation site. Our findings reveal key insights into FOXK2 regulation and the contribution to understanding breast cancer cell biology.

## Graphical abstract

Higher FOXK2 protein levels are associated with increased AKT phosphorylation and chemoresistance in breast cancer cells. AKT modulates FOXK2 protein levels, with no such regulation in FOXK2 mRNA levels. GSK3β, a known substrate of AKT (dotted arrow), does not regulate FOXK2 in our cellular model. Both phosphorylated AKT and FOXK2 are predominantly located in the nucleus of drug‐resistant cells, regardless of AKT phosphorylation. Notably, FOXK2 is a substrate of AKT, and phosphorylation at S398 in FOXK2 seems to contribute to enhanced cell growth, viability, and clonogenic capacity, even in the presence of chemotherapeutic agents. Consistently, breast tumor samples exhibit increased FOXK2 phosphorylation at different sites. The processes indicated in gray do not occur in our cellular model.

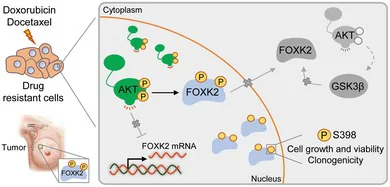

## 1 Introduction

FOXK2 is a transcription factor of forkhead box (FOX) family, whose members share an evolutionarily conserved DNA‐binding domain (FHRE) [1]. In addition, FOXK2 possesses the forkhead‐associated domain (FHA), which mediates interactions with phosphorylated proteins, especially at threonine and serine [2]. FOXK2 is involved in multiple biological processes, such as cell cycle, proliferation, differentiation, apoptosis, DNA repair, autophagy, *de novo* nucleotide synthesis, and metabolism (for review, please see [3]). Growing evidence shows that FOXK2 has a dual function in cancer biology, acting as an oncogene [4, 5, 6, 7, 8, 9, 10, 11, 12] as well as tumor suppressor [13, 14, 15, 16, 17, 18, 19, 20, 21]. In breast cancer, FOXK2 suppresses the proliferation of estrogen receptor‐positive cells [22] and inhibits tumor growth and metastasis in vivo [13]. Interestingly, it has also been implicated in promoting drug sensitivity in a FOXO3‐dependent manner in breast cancer cells. However, chemoresistant cells have constitutively high levels of FOXK2 protein and fail to activate *FOXO3* transcription [23]. These findings suggest that FOXK2 may be regulated at the post‐translational level, resulting in the inactivation of its tumor suppressive function, despite elevated protein levels. This hypothesis is supported by increasing evidence that FOXK2 is modulated by phosphorylation [12, 24, 25, 26], acetylation [27], ubiquitination [28] and SUMOylation [29, 30].

Based on an *in silico* analysis using the iGPS software [31], we identified protein kinase B (PKB also called AKT) as the main possible regulator of FOXK2, with different phosphorylation target sites. Hyperactivation of the PI3K‐AKT pathway is particularly common in breast cancer [32], playing a role in cell survival, proliferation, metabolism, and chemoresistance [32, 33, 34], but its impact on FOXK2 phosphorylation and function has not been demonstrated. Therefore, we hypothesized that FOXK2 could be regulated by AKT‐mediated phosphorylation to modulate survival and drug response in breast cancer cells.

Herein, we have demonstrated that FOXK2 protein levels are modulated in AKT‐depleted or overexpressing cells, with no such modulation in FOXK2 mRNA levels. AKT knockdown also impairs breast cancer cell survival and response to therapeutic treatment, accompanied by alterations in FOXK2 gel mobility. Our results also reveal FOXK2 as a substrate for AKT phosphorylation in breast cancer cell lines. Accordingly, breast tumor samples exhibit higher levels of FOXK2 phosphorylation (e.g., serine residue 398/S398) compared to normal breast tissue. Notably, the phosphodeficient S398A form of FOXK2 impacts clonogenicity and cell survival of breast cancer cells in the presence or absence of chemotherapy drugs. Overall, we have uncovered new evidence on FOXK2 post‐translational regulatory mechanisms, defining a novel functionally significant residue in FOXK2, which has important implications for the AKT‐FOXK2 axis in breast cancer biology and treatment.

## 2 Materials and Methods

### 2.1 Cell Culture and Chemicals

The human ductal invasive breast cancer cell lines MCF‐7 and MDA‐MB‐231 were used as models in our study. The cell lines were cultured in Dulbecco's modified Eagle's medium (DMEM, Gibco, Thermo Fisher Scientific, Waltham, MA, USA) supplemented with 10% inactivated fetal bovine serum (FBS, Gibco) and penicillin/streptomycin (10 μL/mL). The cells were maintained in a humidified incubator with 5% CO_2_ at 37°C and were trypsinized (Trypsin 0.125%, Sigma‐Aldrich, St. Louis, MO, USA) prior to the experiments. The cell lines were genotyped by short tandem repeats, and mycoplasma contamination was periodically monitored.

The cells were treated with different concentrations of the chemotherapeutic drugs doxorubicin (DOX; Fauldoxo, Libbs, São Paulo, Brazil) and docetaxel (DTX; Glenmark, Mumbai, India). Serial dilutions of drug stock solutions (DOX: 1.72 mM in 0.1% NaCl solution; DTX: 1 mM in culture medium) were performed in culture medium prior to the experiments. Drug treatments were carried out in 48 and 72 h. Also, clinical concentrations of DOX (1 μM) and DTX (100 nM) were included in the assays [35, 36]. For proteasome inhibition, the MG132 compound (M7449, Sigma‐Aldrich) was used at the concentration of 10 μM. Cells transfected with FOXK2 genetic variants were treated with the MG132 proteasome inhibitor for 6 h prior to cell lysis.

### 2.2 Plasmids

The plasmids encoding human FOXK2 pCMV5‐FOXK2WT and the empty vector pCMV5 were previously described [24]. The S398A and S398E mutations were introduced by site‐directed mutagenesis by overlap extension using the pCMV5‐FOXK2WT plasmid as template. The final PCR products were then digested with KpnI and XbaI and ligated into equally digested pFLAG‐CMV. All mutations were confirmed by sequencing. Primers used for mutagenesis and sequencing are listed in Tables S1 and S2. The pBABE‐(myr)Akt:ER conditional expression vector encodes a constitutively active version of myristoylated AKT induced by 4‐hydroxytamoxifen (4‐OHT; Sigma‐Aldrich, H6278).

### 2.3 Transient Transfections

Plasmid transfections were performed with Lipofectamine 2000 (Life Technologies, Thermo Fisher Scientific) in the presence of OPTI‐MEM (OPTI‐MEM I Reduced Serum Media, Gibco), according to manufacturers' instructions. Briefly, cells were plated and left to adhere for 24 h, after which cells were transfected using a 1:1 ratio of DNA to Lipofectamine 2000. After 24 h, the cells were collected for protein analysis by Western blotting and seeded for drug treatments. For the pBABE‐(myr)Akt:ER, 24 h after transfection, the cells were treated with 4‐OHT for 24 h.

### 2.4 Pharmacological Inhibition of AKT

To directly inhibit AKT activity, we used MK‐2206, an allosteric inhibitor which stabilizes AKT in its inactive conformation. To establish the toxicity profile of MDA‐MB‐231 cells in response to this compound, 10^4^ cells were plated in 96‐well plates and left to adhere for 24 h. Then, cells were treated with DMSO (vehicle) or increasing concentrations of MK‐2206 and were maintained in culture for an additional 8, 16, 24, and 48 h prior to the MTT assay. To evaluate protein levels, MDA‐MB‐231 cells were plated and left to adhere for 24 h. Afterwards, cells were treated with DMSO or 25 μM MK‐2206 for 24 h, and total and fractionated protein extracts were analyzed by Western blotting. To assess the effect of MK‐2206 treatment on FOXK2 ectopic levels, cells were transfected with either pCMV5 or pCMV5‐FOXK2WT plasmids and treated with 10 μM MK‐2206. After 24 h, cells were collected for western blot analysis.

### 2.5 Gene Inhibition by siRNAs

For AKT1 and GSK3β knockdown, cells were transfected with 10 nM validated siRNAs using Lipofectamine RNAimax (Life Technologies, Thermo Fisher Scientific, Waltham, MA, USA) in the presence of OPTI‐MEM (OPTI‐MEM I Reduced Serum Media, Gibco, Thermo Fisher Scientific) based on manufacturer's recommendations. The siRNAs used were as follows: siRNA AKT1 (AM16704) and non‐silencing control (NSC) siRNA (4 390 847; Ambion Product List, Thermo Fisher Scientific); siRNA GSK3β (QIAGEN, Hilden, Germany). For the last siRNA, cells were exposed to the ‘Mock’ condition (all transfection reagents, except RNA) as a control. After 24, 48 or 72 h, cells were collected for Western blot analysis and seeded for drug treatments.

### 2.6 Co‐Transfection Protocol

To evaluate the effect of AKT gene inhibition on ectopic FOXK2 protein levels, and the impact of modulating both proteins on chemosensitivity, we performed co‐transfection experiments using the methodologies described in the two previous sections. Briefly, MDA‐MB‐231 cells were transfected with either NSC or AKT1 siRNA for 48 h. Subsequently, cells were transfected with pCMV5 or pCMV5‐FOXK2WT plasmids [24]. After 24 h, cells were collected for protein analysis by Western blotting and seeded for drug treatment assays.

### 2.7 Western Blotting

Whole‐cell extracts were prepared in lysis buffer (FNN0011, Invitrogen, Thermo Fisher Scientific) according to the manufacturer's protocol. Protease (P8340, Sigma‐Aldrich, St. Louis, MO, USA) and phosphatase (04906837001, PhosSTOP EASYpack, Roche, Basel, Switzerland) inhibitors were added to the lysates and protein quantification was performed using the BioRad Kit (Hercules, CA, USA), based on the Lowry method [37]. The protein samples were prepared with loading buffer (115 mM Tris–HCl pH 6.8; 15% SDS; 10% glycerol; 100 mM β‐mercaptoethanol; 0.1% blue bromophenol) and denatured at 100°C for 5 min. A total of 15–30 μg of proteins were subjected to electrophoresis in 7 or 10% polyacrylamide gel (SDS‐PAGE) or 7.5% SuperSep Phos‐tag SDS‐PAGE (198‐17981, FUJIFILM Wako Pure Chemical Corporation, Osaka, Japan). Electrophoresis was performed in running buffer (25 mM Tris, 192 mM Glycine and 0.1% SDS; pH 8.3; Bio‐rad) at 120 V. The Phos‐tag gels were submitted to a pre‐treatment before transfer: three incubations with transfer buffer plus 10 mmol/L EDTA for 20 min each with buffer exchanges and one incubation with transfer buffer without EDTA for 10 min, both with gentle agitation. Then, proteins were transferred to nitrocellulose membranes (GE healthcare, Chicago, IL, USA) using a wet system (Transfer buffer: 25 mM Tris, 192 mM Glycine and 20% methanol; pH 8.3; Bio‐rad) at 100 V for 2 h. Proteins were stained with Ponceau Red and membranes were blocked for 1–2 h with 5% low‐fat milk diluted in Tris buffered saline (TBS) plus 0.05% Tween. Then, membranes were incubated at 4°C overnight with the primary antibodies: FOXK2 (A301‐729A, 1:500), and Hsc70 (sc‐7298, 1:1000) were purchased from Bethyl Laboratories (Montgomery, TX, USA) and Santa Cruz Biotechnology (Dallas, TX, USA), respectively. Phospho‐AKT (9271, 1:500), total AKT (9272, 1:1000), Phospho‐AKT Substrate (RXRXXS*/T*) (23C8D2) (10 001, 1:1000), Ubiquitin (3936, 1:1000), Phospho‐GSK3β (9336, 1:1000) and GSK3β total (9315, 1:1000) were obtained from Cell Signaling (Danvers, MA, USA). GAPDH (G8795, 1:10000) and β‐actin (A5441, 1:10000) were purchased from Sigma‐Aldrich. Primary antibodies were detected using horseradish peroxidase‐linked anti‐mouse or anti‐rabbit conjugates (Sigma‐Aldrich) and visualized using the Clarity Max Western ECL Kit (BioRad). The proteins Hsc70, GAPDH and β‐actin were used as constitutive controls. The protein bands were detected using a digital system (ChemiDoc Imaging System, BioRad).

### 2.8 Subcellular Fractionation

The cytoplasmic and nuclear fractions were isolated from untransfected MCF‐7 and MDA‐MB‐231 cell lines, as well as MCF‐7 cells transfected with FOXK2 genetic variants, using the NE‐PER kit (NEPER Nuclear and Cytoplasmic Extraction Reagents, Thermo Fisher Scientific). The protein levels of FOXK2, phospho‐AKT, and total AKT were evaluated in subcellular fractions by Western blotting. Lamin B (sc‐374015, 1:1000, Santa Cruz Biotechnology) protein was used as a nuclear constitutive control, while GAPDH (G8795, 1:10000, Sigma‐Aldrich) was used as a cytoplasmic constitutive control.

### 2.9 Immunofluorescence

To assess the subcellular localization of FOXK2 genetic variants, cells were fixed with 4% paraformaldehyde, washed with phosphate buffered saline (PBS) and incubated in a blocking solution of 1% bovine serum albumin (BSA) and 0.3% Triton X‐100 (in PBS pH 8.0) for 30 min. Subsequently, the cells were incubated in 50 mM NH4Cl for 15 min and remained in slides with the primary antibody anti‐Flag (1:100; Sigma‐Aldrich) at 4°C overnight. After this period, the cells were incubated with the secondary antibody (1:500 Alexa Fluor 488, Invitrogen) for 1 h at 4°C and stained with the DAPI nuclear marker (1:1000) for 10 min, when the slides were fixed with N‐propyl gallate. The cells were visualized with an Axiovert 100 microscope (Carl Zeiss, Germany) coupled to an Olympus DP71 high‐resolution camera (Olympus, Japan).

### 2.10 Measurement of Metabolic Cell Viability

To assess the metabolic cell viability following drug treatment, we performed the MTT assay. Briefly, 10^4^ cells were plated in 96‐well plates and left to adhere for 24 h, after which they were treated with MK‐2206 for 8, 16, 24 and 48 h or DOX or DTX for 48 and 72 h. Four hours before the end of these periods, the MTT was added and the plates were incubated (5% CO_2_ at 37°C) protected from light. Afterwards, the plates were centrifuged (915 g, 3 min), the supernatant was removed, and formazan crystals were dissolved in DMSO. The optical density was obtained by spectrophotometry (SpectraMax Plus384) at 492 nm. All experiments were performed in independent triplicates and the results were calculated using metabolic cell viability means.

### 2.11 Clonogenic Assay

The clonogenic assay was used to evaluate the long‐term colony formation capacity. For this, 2000 cells were plated in six‐well plates and left to adhere for 24 h, after which cells were treated with increasing concentrations of DOX. After 48 h of incubation, the culture medium was replaced with fresh drug‐free medium and the cells were observed until colony formation, for approximately 14 days. This assay was also carried out using untreated cells. Colonies were washed with phosphate‐buffered saline (PBS) and fixed with absolute ethanol for 15 min at room temperature. After three more washes with PBS, the colonies were stained with 0.5% crystal violet for 1 h, washed with flowing water and air‐dried. Finally, the colonies were photographed and the crystal violet was solubilized in 1 mL of 33% acetic acid. The optical density was obtained by spectrophotometry (SpectraMax Plus384) at 595 nm. All experiments were performed in independent triplicates and the results were calculated using long‐term cell viability means.

### 2.12 Phosphatase Assay

Cells were lysed by sonication in Triton Lysis Buffer (TLB). Afterwards, 100 μg of protein were incubated in a total of 50 μL containing 400 units of lambda protein phosphatase (λPP; P0753S, New England BioLabs Inc., Ipswich, MA, USA), 1× PMP (NEBuffer Pack for Protein MetalloPhosphatases; B0761S, New England BioLabs Inc.) and 1 mM MnCl_2_ (B1761S, New England BioLabs Inc.) for 1 h at 37°C. Protein bands were evaluated by western blotting.

### 2.13 Immunoprecipitation Assays

For immunoprecipitation of endogenous FOXK2 protein, the cells were lysed in Mild‐RIPA buffer (Tris 50 mM pH 7,4, NaCl 150 mM, EDTA 1 mM e NP‐40 1% v/v) with protease and phosphatase inhibitors on ice for 30 min. Cell debris was removed by centrifugation at 16000×*g*, 4°C for 10 min and the soluble fractions were collected. About 3–4 mg of the whole‐cell extract were then incubated overnight with A/G PLUS‐Agarose (sc‐2003; Santa Cruz Biotechnology, Dallas, TX, USA) coupled with the primary antibodies at 4°C (FOXK2, 12 008; Phospho‐AKT, 9271, Cell Signaling, Boston, MA, USA). Since caspase‐1 and FOXK2 are not reported to interact, resins conjugated to anti‐caspase‐1 antibody (sc‐515, Santa Cruz Biotechnology) were used as a negative control. The agarose beads were washed four times with Mild‐RIPA buffer and boiled in SDS loading buffer (100°C, 5 min). The immunoprecipitates were analyzed by Western blotting.

For immunoprecipitation of ectopic FOXK2 protein, constructs encoding Flag‐tagged FOXK2 were transiently transfected into MCF‐7 cells. The transfected cells were lysed with Mild‐RIPA buffer containing protease and phosphatase inhibitors following the protocol mentioned above. The soluble fractions were incubated overnight at 4°C with A/G PLUS‐Agarose coupled with FLAG antibody (F1804, Sigma‐Aldrich). The agarose beads were washed four times with Mild‐RIPA buffer and boiled in SDS loading buffer (100°C, 5 min). The immunoprecipitates were analyzed by Western blotting.

### 2.14 Real‐Time Reverse Transcription Polymerase Chain Reaction (RT‐qPCR)

The extraction of total RNA was performed by Trizol reagent (TRIzol, Invitrogen, Thermo Fisher Scientific) according to the manufacturer's instructions. RNA concentration and purity were measured using NanoDrop 1000 (Thermo Scientific). Complementary DNA (cDNA) was synthesized from 3 μg RNA using the SuperScript II First‐Strand and RT Random Primers (Invitrogen, Thermo Fisher Scientific). For real‐time quantitative PCR (qRT‐PCR), SYBR Green PCR Master Mix kit (Applied Biosystems; Thermo Fisher Scientific) was used according to the manufacturer's instructions. All PCR assays were done in triplicate using the 7500 Real‐Time PCR System (Applied Biosystems; Thermo Fisher Scientific). The cycling program was 95°C for 20 min followed by 40 cycles of 95°C for 15 s and 60°C for 1 min. The 2^−∆CT^ method was applied to quantify *FOXK2* expression, using *GAPDH* as an endogenous reference. The forward and reverse primers used were FOXK2‐F: 5′‐AGTCTCCAGTGAAGGCCGTA‐3′ and FOXK2‐R: 5′‐CCCACCTTGTACCCTGAAGA‐3′ and GAPDH‐F: 5′‐ACCCACTCCTCCACCTTTGA‐3′ and GAPDH‐R: 5′‐CTGTTGCTGTAGCCAAATTCGT‐3′.

### 2.15 Analysis of FOXK2 Protein Levels and Phosphorylation in Breast Cancer Patient Sample Database

To evaluate the protein levels and phosphorylation profile of FOXK2 in breast cancer, we analyzed the proteomics and phosphoproteomics data from the Clinical Proteomic Tumor Analysis Consortium (CPTAC) [38, 39], through the UALCAN platform, an online tool available at: <http://ualcan.path.uab.edu/analysis‐prot.html> [40, 41]. In this analysis, total and phosphorylated FOXK2 protein levels were compared among 125 samples of primary breast tumors and 18 samples of normal breast tissue adjacent to the tumor [38]. The protein level values were transformed to log_2_ ratio (individual sample/common reference sample). The reference sample was constructed from the protein pool of 40 tumor samples representative of the cohort analyzed in the study [42]. The data were also normalized among the samples expressed as Z‐value.

### 2.16 Statistical Analysis

Data are presented as mean ± standard error of the mean (SEM) from at least three independent experiments. Statistical analyses were performed with GraphPad Prism (GraphPad Software Inc.) using Student's *t*‐test, One‐Way or Two‐Way ANOVA, as appropriate. Values of *p* ≤ 0.05 were considered statistically significant.

## 3 Results

### 3.1 AKT Phosphorylation Levels Associate With FOXK2 Protein Content and Breast Cancer Drug Resistance and Survival

We have previously demonstrated that poor prognosis breast cancer patients and drug‐resistant ductal invasive breast cancer cell line models present constitutively high protein levels of FOXK2, which fail to activate *FOXO3a* gene expression [23]. These findings suggest that posttranslational modifications might impair FOXK2 functions, contributing to drug resistance and unfavorable clinical outcomes. To address this issue, we have initially explored the potential role of FOXK2 phosphorylation. Based on a prediction of site‐specific kinase‐substrates, we have found the oncogenic kinase AKT (isoform 1) as the main possible regulator of FOXK2 (Figure 1A) with several putative AKT‐specific phospho‐sites in FOXK2 protein sequence (Figure 1B). Then, we compared AKT phosphorylation and FOXK2 protein levels in MCF‐7 (luminal A subtype, drug sensitive) and MDA‐MB‐231 (triple‐negative subtype; drug‐resistant) cell lines. Our results indicate that the increased protein content of FOXK2 was associated with higher levels of AKT phosphorylation (Figure 1C) and doxorubicin resistance (Figure 1D). To validate the role of AKT1 in our model, we knocked down AKT1 expression in MDA‐MB‐231 cells using small interfering RNA (siRNA) and assessed drug response profiles by short‐ (MTT) and long‐term (clonogenic) cell viability assays. AKT1‐inhibited cells (Figure 1E) had impaired clonogenicity (Figure 1F) and viability in the presence of doxorubicin (Figure 1G). These results validate AKT's role in cell survival in drug‐resistant breast cancer cells.

**FIGURE 1:**
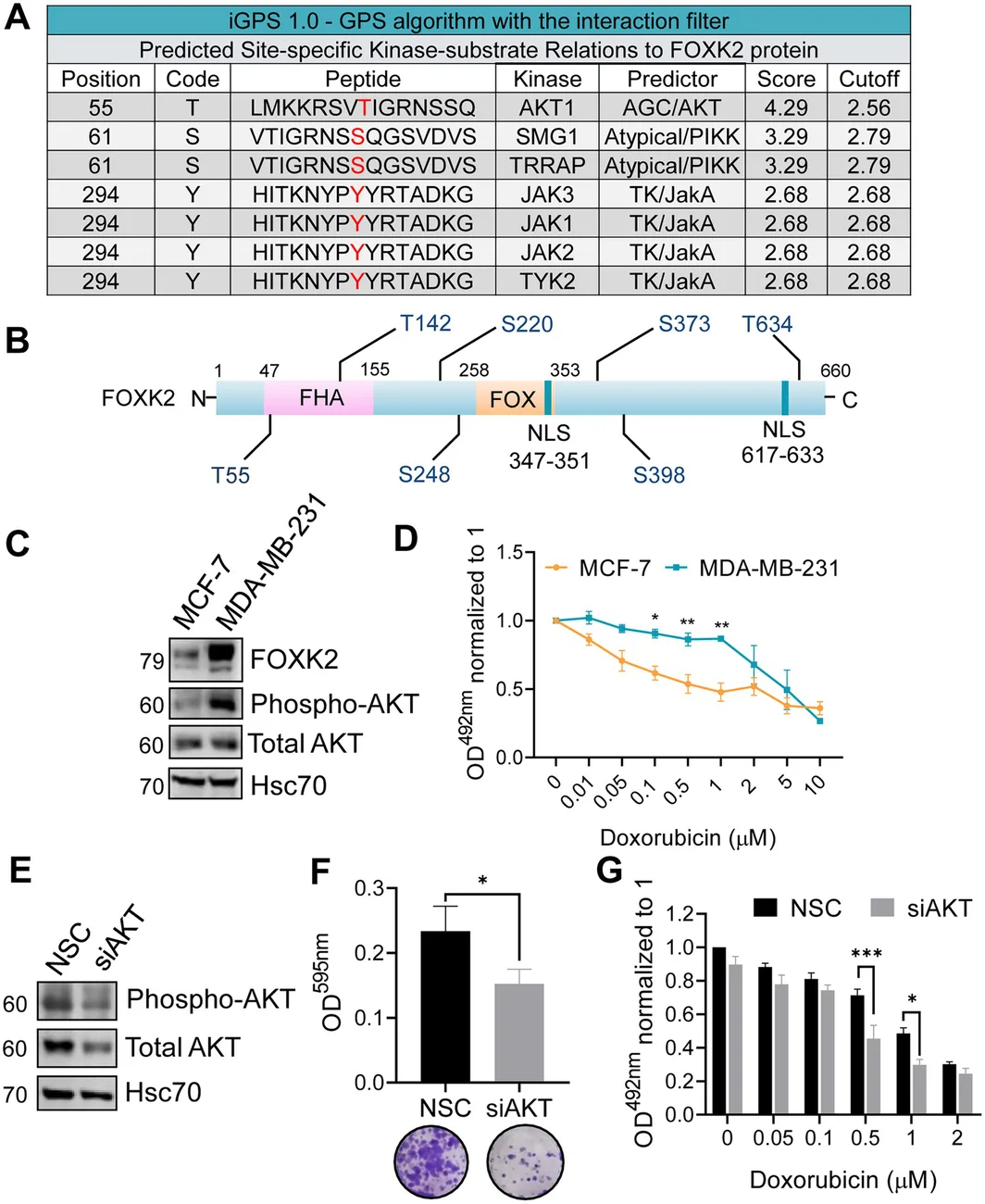
AKT oncogenic kinase as the main possible regulator of FOXK2. (A) FOXK2 consensus sequences for phosphorylation motifs identified through the iGPS web server. (B) Putative AKT1‐specific phospho‐sites in FOXK2 protein sequence. (C) Immunoblotting analyses using MCF‐7 and MDA‐MB‐231 cells. (D) MTT assays were carried out using MCF‐7 and MDA‐MB‐231 cells treated with increasing concentrations of doxorubicin (DOX) for 72 h. (E) Immunoblotting analyses using MDA‐MB‐231 cells transfected with non‐silencing control (NSC) or AKT1 siRNA. (F and G) Colony formation (F) and MTT (G) assays were carried out using siRNA‐transfected cells as described in (E) treated or not with increasing concentrations of DOX for 72 h. Statistical significance was determined by Two‐Way ANOVA or Student's *t*‐test (F) (*p ≤ 0.05; **p ≤ 0.01; ***p ≤ 0.001, significant).

### 3.2 AKT Inhibition Modulates FOXK2 Protein at Both Endogenous and Ectopic Levels in Drug‐Resistant Breast Cancer Cells

Next, we investigated whether AKT inhibition impacts FOXK2 protein content. Our results show that siRNA‐based AKT genetic knockdown resulted in decreased levels of endogenous FOXK2 in MDA‐MB‐231 cells (Figure 2A). AKT is known to phosphorylate GSK [43, 44], which in turn phosphorylates FOXK1/2 in mouse embryonic fibroblasts and liver cell models [25, 45]. To determine whether FOXK2 regulation by AKT is mediated by GSK, we inhibited GSK3β expression using siRNA in MDA‐MB‐231 cells. From the blots, we observed that GSK3β inhibition does not modulate FOXK2 levels either in 24 or 48 h post‐transfection (Figure S1). These results indicate that GSK3β may not regulate FOXK2 protein levels in our cellular model, suggesting that the AKT‐GSK3‐FOXK2 axis could exert its regulatory functions in a cell type specific manner. To further explore the regulation of FOXK2 by AKT, we have made use of MK‐2206, a specific allosteric inhibitor of AKT, particularly AKT1 and AKT2. First, we characterized the toxicity profile of MDA‐MB‐231 cells in response to MK‐2206 by the MTT assay. We observed that cells treated with 5 and 10 μM showed increased viability over time, similarly to the control and DMSO groups, while cells treated with 25 and 50 μM exhibited a significant reduction in viability (Figure S2A). In a time‐course exposure, we have found that 25 and 50 μM MK‐2206 greatly affected metabolic cell viability from 8 to 48 h of treatment (Figure S2B). As a proof of concept, we analyzed AKT phosphorylation following MDA‐MB‐231 cell treatment with 25 μM MK‐2206 and found that reduction in phospho‐AKT can be detected as early as 4 h of exposure (Figure S3). Based on these results, we used 25 μM MK‐2206 as the lowest effective concentration known to inhibit AKT phosphorylation and observed that FOXK2 endogenous levels were modulated following AKT allosteric inhibition for 24 h (Figure 2B).

**FIGURE 2:**
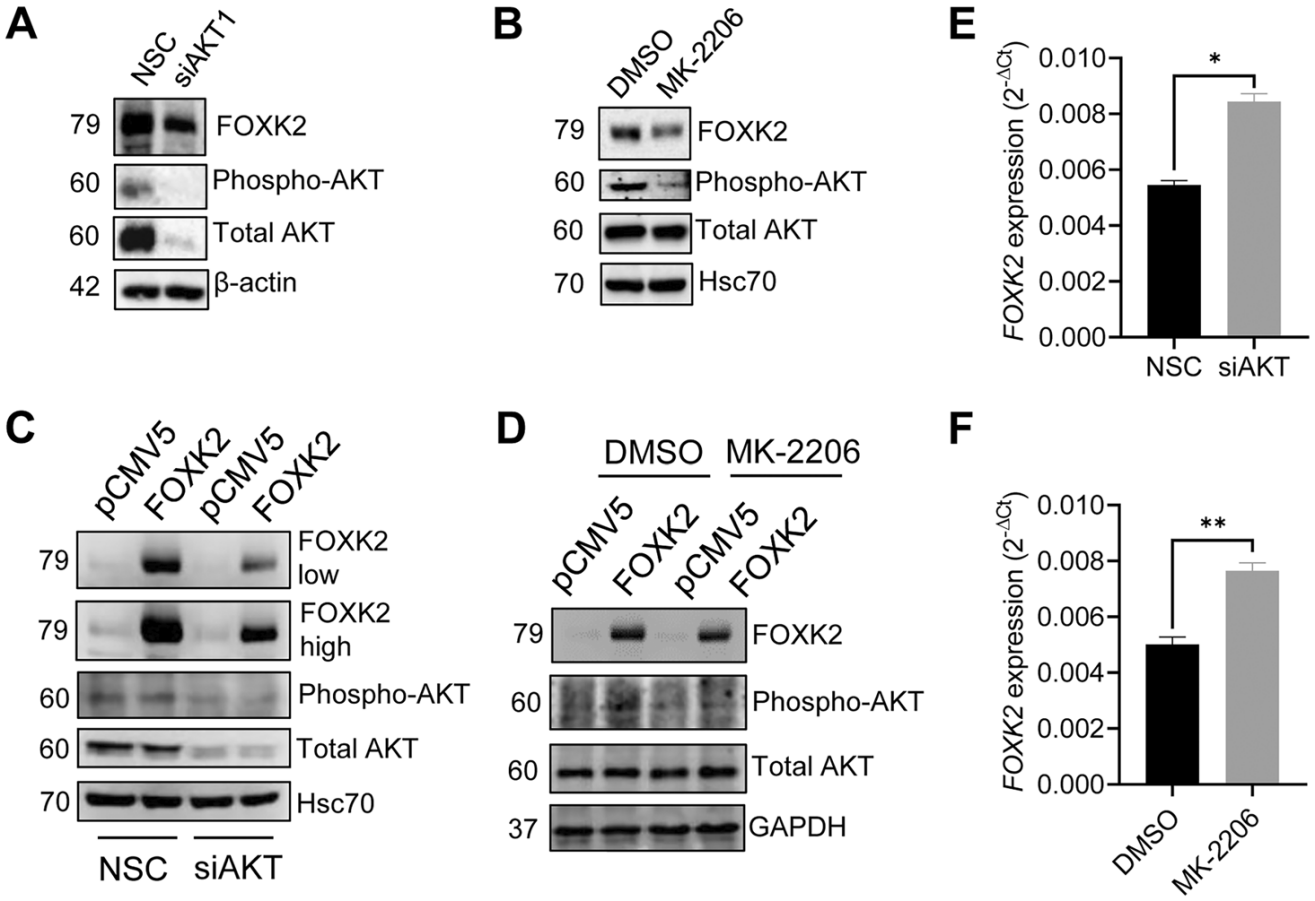
AKT inhibition modulates endogenous and exogenous protein levels of FOXK2. (A and B) Immunoblotting analyses using MDA‐MB‐231 cells transfected with non‐silencing control (NSC) or AKT1 siRNA for 72 h (A) or treated with DMSO vehicle control or 25 μM MK‐2206 for 24 h (B). (C) Immunoblotting analyses using MDA‐MB‐231 cells transfected with NSC or AKT1 siRNA for 48 h and co‐transfected with the empty (pCMV5) or the wild‐type FOXK2 vector for 24 h. (D) Immunoblotting analyses using MDA‐MB‐231 cells transfected with the pCMV5 or the FOXK2 vector and treated with DMSO or 10 μM MK‐2206 for 24 h. (E and F) Quantification of RNA levels by real‐time quantitative PCR (SYBR Green Master Mix method) of MDA‐MB‐231 cells with AKT inhibition as described in (A and B), respectively. Statistical significance was determined by Student's *t*‐test (*p ≤ 0.05; **p ≤ 0.01, significant).

We also evaluated the subcellular localization of FOXK2 and AKT in breast cancer cells. Corroborating data in literature, we have found that FOXK2 is predominantly localized in the nucleus of MCF‐7 and MDA‐MB‐231 cells (Figure S4). Despite their cytoplasmic expression of total AKT, MDA‐MB‐231 cells have shown higher levels of nuclear phosphorylated AKT than MCF‐7 cells (Figure S4). These data suggest that FOXK2 and phosphorylated AKT are predominantly found at the same subcellular compartment in MDA‐MB‐231 cells, which reinforces our hypothesis that these proteins can functionally interact to contribute to chemoresistance.

Then, we questioned whether AKT kinase could modulate FOXK2 ectopically expressed in MDA‐MB‐231 cells. For this purpose, we induced FOXK2 overexpression concomitant with AKT‐knockdown and found that AKT1 inhibition modifies not only endogenous but also ectopic expression of FOXK2, particularly slower mobility protein bands (Figure 2C). Interestingly, siRNA‐based AKT knockdown reduced cell growth and increased sensitivity to doxorubicin regardless of FOXK2 levels (Figure S5). These findings indicate that the modulation of AKT is sufficient to modulate chemosensitivity in drug‐resistant breast cancer cells. Next, we performed experiments involving FOXK2 overexpression combined with MK‐2206 treatment, which revealed that FOXK2 ectopic levels were slightly reduced following AKT allosteric inhibition (Figure 2D). This lesser reduction might have occurred due to less pronounced effects of MK‐2206 on AKT inhibition compared to those obtained through siRNA‐based AKT1 knockdown.

We next performed genetic and pharmacological inhibition of AKT in MDA‐MB‐231 cells to investigate FOXK2 band mobility on Phos‐tag SDS‐PAGE gels, which separate phosphorylated from nonphosphorylated proteins. Considering the predicted molecular weight of 79 kDa for FOXK2, we have observed that FOXK2 migrates with slower mobility bands in Phos‐tag gels (Figure S6), suggesting that it can be regulated by phosphorylation. Then, we observed that AKT inhibition by MK‐2206 (Figure S6A) and a siRNA‐based technique (Figure S6B,C) have led to a global reduction in FOXK2 phospho species of different molecular weights.

To exclude the possibility that reduced FOXK2 protein content mediated by AKT inhibition could involve FOXK2 gene downregulation, we also analyzed FOXK2 transcript levels. Notably, the inhibition of AKT by both genetic (Figure 2E) and pharmacological (Figure 2F) means leads to an increase rather than a reduction in FOXK2 transcript levels, reinforcing that FOXK2 is regulated by AKT at the post‐translational level. Altogether, these findings suggest that AKT inhibition results in a decrease of FOXK2 protein at both endogenous and ectopic levels in drug‐resistant breast cancer cells.

### 3.3 Overexpression of Constitutively Active AKT Improves FOXK2 Protein Content in Drug‐Sensitive Breast Cancer Cells

To confirm this data, we have made use of a conditional expression system in which constitutively active myristoylated AKT (myrAKT) is expressed following treatment with 4‐hydroxytamoxifen (Figure 3A). Our results show that 4‐hydroxytamoxifen‐induced AKT constitutively active expression increased FOXK2 protein content in drug‐sensitive MCF‐7 cells (Figure 3B) while effects were less pronounced in drug‐resistant MDA‐MB‐231 cells (Figure 3C), which exhibit higher FOXK2 basal protein levels. To exclude the possibility that 4‐hydroxytamoxifen by itself could modulate FOXK2 levels, we also used untransfected cells exposed to this compound as experimental controls. We observed no increase in FOXK2 protein content in 4‐hydroxytamoxifen‐treated cells in the absence of myrAKT (Figure S7). Consistently, we have verified an increase in FOXK2 phospho species in Phos‐tag gels following overexpression of constitutively active AKT in MCF‐7 cells (Figure S8). These findings indicate that AKT overexpression increases FOXK2 protein content in breast cancer cells.

**FIGURE 3:**
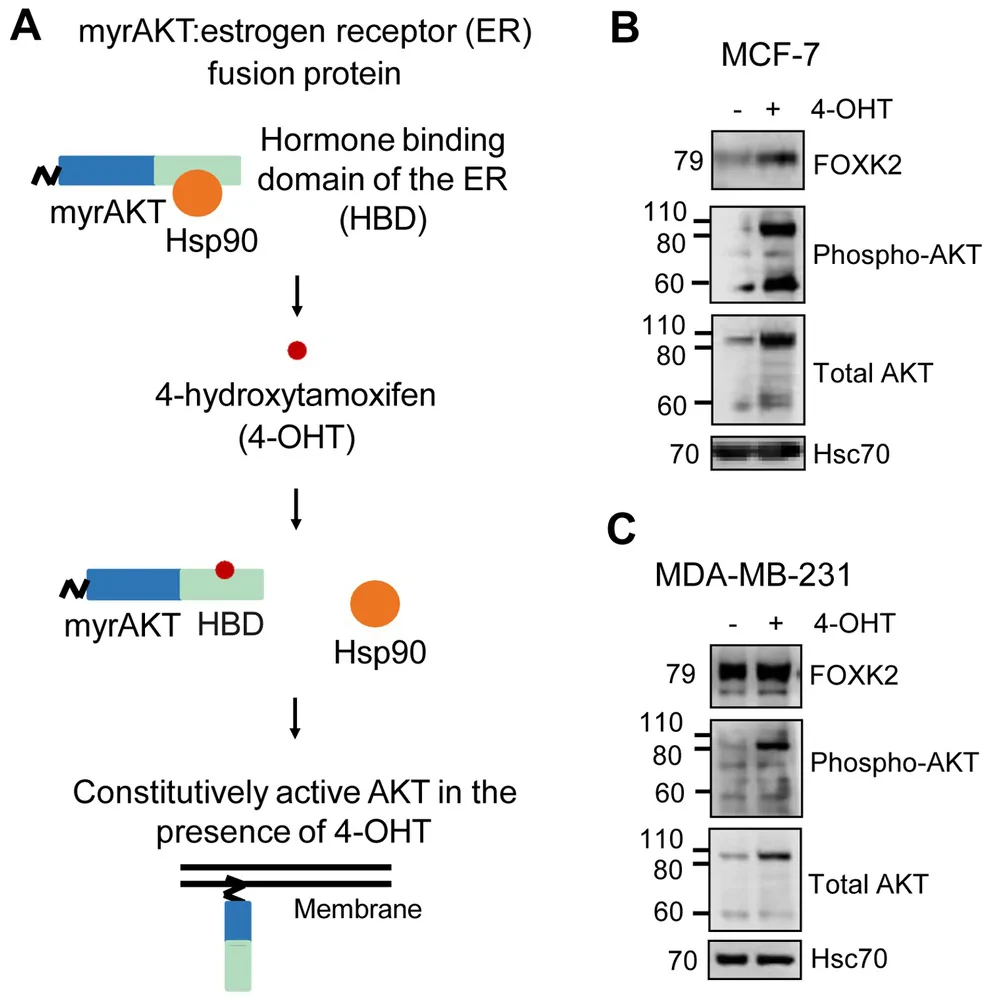
Overexpression of a constitutively active myristoylated AKT increases FOXK2 protein content. (A) pBABE‐(myr)Akt:ER vector operating scheme. (B and C) Immunoblotting analyses using MCF‐7 (B) and MDA‐MB‐231 (C) cells transfected with pBABE‐(myr)Akt:ER vector and treated with 200 nM 4‐hydroxytamoxifen (4‐OHT) or ethanol vehicle control for 24 h.

### 3.4 FOXK2 Is Found Phosphorylated in Breast Cancer Cellular Models and Patient Samples

Considering that the putative AKT‐FOXK2 regulatory axis implicates FOXK2 phosphorylation by AKT, we performed experiments involving lambda protein phosphatase (λPP) to verify phosphorylation‐dependent mobility shifts on Western blot. Treatment with λPP has changed the mobility of the FOXK2 protein band, which migrates faster in the gel (Figure 4A), denoting phosphate group removal by the enzyme. Next, we have investigated if FOXK2 is found phosphorylated in breast cancer patients' samples. Using the proteomics and phosphoproteomics data of the Clinical Proteomic Tumor Analysis Consortium (CPTAC) [38], we have found lower total protein content of FOXK2 among tumor, compared to non‐tumor samples, although it did not reach statistical significance (Figure 4B). Nevertheless, FOXK2 phosphorylation levels are higher in breast tumors than in normal adjacent breast tissues (Figure 4C–F). Except for serine 239 (S239) (Figure 4C), differences in FOXK2 phosphorylation levels have reached statistical significance for serine 170 (S170) (Figure 4D), serine 428 (S428) (Figure 4E) and importantly, the putative AKT1 phosphorylation site serine 398 (S398) (Figure 4F). Together, these findings show that breast cancer exhibits high levels of FOXK2 phosphorylation.

**FIGURE 4:**
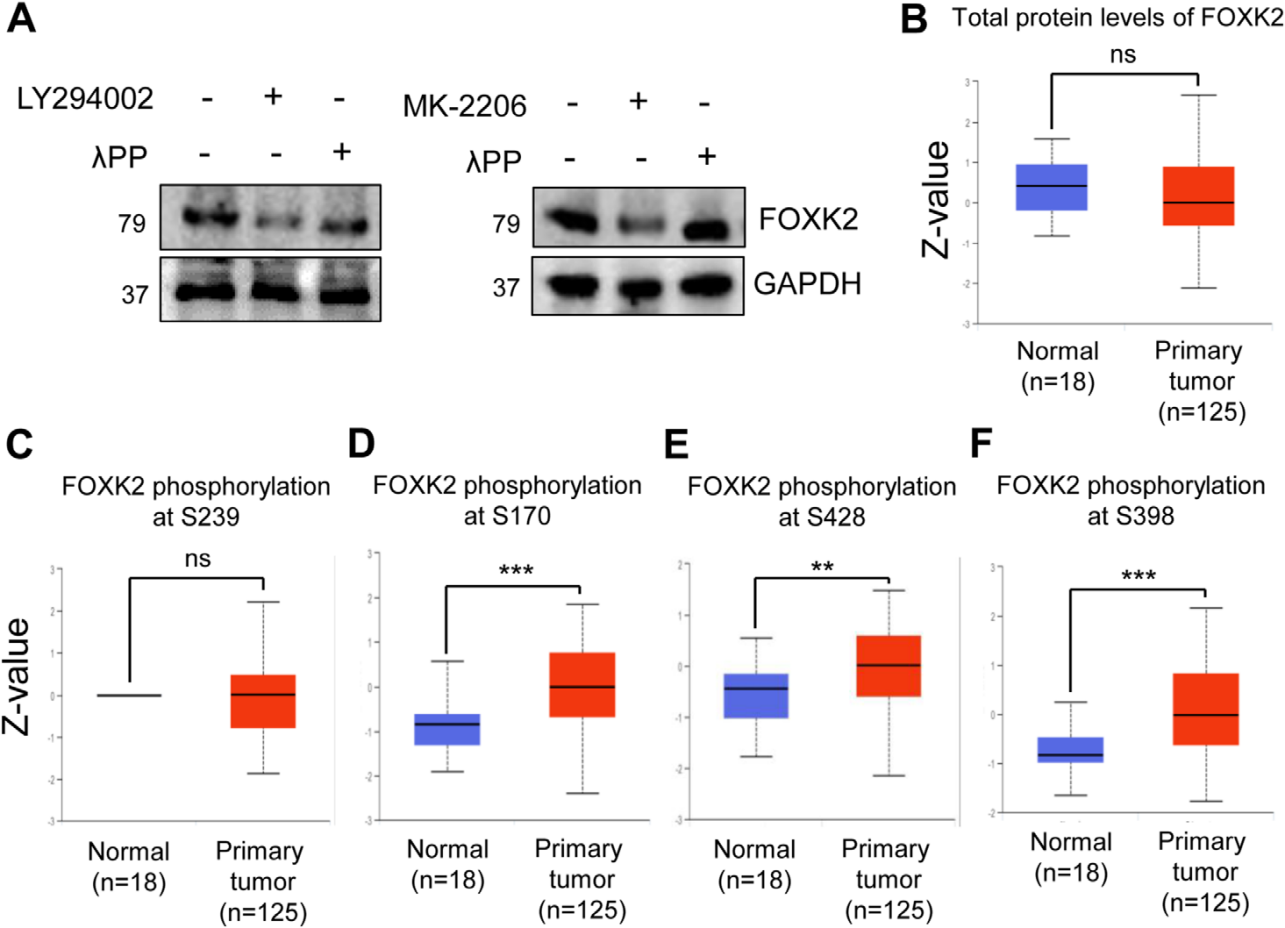
FOXK2 is phosphorylated in breast cancer patient samples and cellular models. (A) Immunoblotting analyses using MDA‐MB‐231 treated with DMSO vehicle control or 30 μM LY294002 for 1 h (left panel) or 25 μM MK‐2206 for 24 h (right panel) and exposed (+) or not (−) to lambda protein phosphatase (λPP) for 1 h at 37°C. (B–F) Analysis of total (B) and phosphorylated FOXK2 levels at serine (S239) (C) serine 170 (S170) (D) serine 428 (S428) (E) and serine 398 (S398) (F) in primary breast cancer tumor samples (*n* = 125) and normal tissue samples adjacent to the tumor (*n* = 18), using data from the Clinical Proteomic Tumor Analysis Consortium (CPTAC), available at UALCAN (http://ualcan.path.uab.edu). Statistical significance was determined by Student's t‐test, using log transformed values (base 2) (ns, not significant; **p ≤ 0.01; ***p ≤ 0.001, significant).

### 3.5 FOXK2 Mutation at Serine398 Impairs Breast Cancer Cell Growth and Viability

Next, we explored phosphorylation sites in the FOXK2 protein sequence through the analysis of data from PhosphoSitePlus (https://www.phosphosite.org/proteinAction.action?id=16478), an online resource for investigating post‐translational modifications predicted from published literature and high‐throughput experiments in human and mice [46]. Figure 5A reveals that multiple phosphorylation sites could be predicted in FOXK2 protein, some of which have also been found deregulated in breast cancer patients (purple numbers and Figure 4C–E) and predicted as putative for AKT phosphorylation (red numbers and Figure 1B). Notably, serine 398, a previously uncharacterized phosphorylation site on FOXK2, has drawn our attention as the only FOXK2 residue identified in all three strategies approached (green number and Figures 1B, 4F, 5A). We therefore selected this for single site‐directed mutagenesis. Following the construction of phosphodeficient S398A and phosphomimic S398E FOXK2, we transfected MCF‐7 cells with FOXK2 genetic variants and compared FOXK2 protein content. Mutating S398 for alanine leads to FOXK2 reduced protein levels, an effect that could be reversed in cells transfected with S398E (Figure 5B).

**FIGURE 5:**
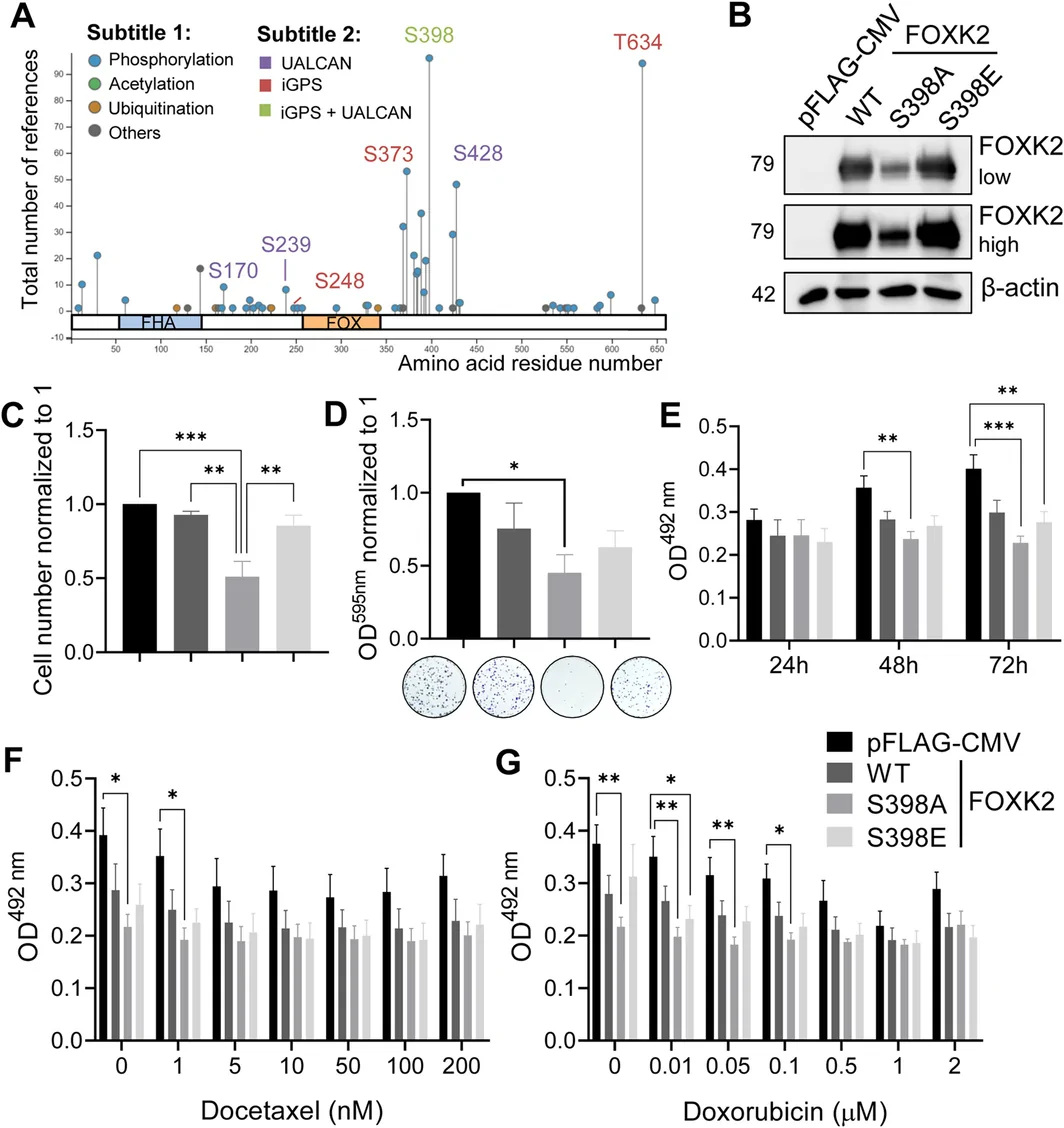
FOXK2 mutation at Serine398 impairs breast cancer cell growth and viability. (A) FOXK2 consensus sequences for phosphorylation motifs using data from the iGPS web server, the PhosphoSitePlus and the CPTAC, available at UALCAN. (B) Immunoblotting analyses using MCF‐7 cells transfected with the empty vector (pFLAG‐CMV), wild‐type, S398A or S398E FOXK2 variants for 24 h. (C) Cell count with trypan blue exclusion 24 h after transfection described in (B). (D and E) Colony formation (D) and MTT (E) assays were carried out using transfected cells as described in (B). (F and G) MTT assays were carried out using transfected cells as described in (B) treated with increasing concentrations of docetaxel (F) or doxorubicin (G) for 72 h. Statistical significance was determined by One‐Way or Two‐WTTay ANOVA. All expression vectors were compared within each treatment concentration and only significant comparisons are shown (*p ≤ 0.05; **p ≤ 0.01; ***p ≤ 0.001, significant).

To investigate FOXK2 subcellular localization upon overexpression, we transfected cells with FOXK2 genetic plasmids and extracted proteins from both cytoplasmic and nuclear fractions. As shown in Figure S9A, FOXK2 localization remains predominantly nuclear within all genetic variants, with relative protein abundance varying similarly to total extracts. The wild type and S398E forms of FOXK2 also exhibit FOXK2 in the cytoplasm, probably due to very high protein levels. To confirm these results, we stained cells transfected with FOXK2 genetic variants with anti‐Flag antibody. Immunofluorescence experiments indicate that FOXK2 overexpressing cells exhibit a diffuse pattern of FOXK2 staining in all genetic variants (Figure S9B). Although perinuclear staining can be observed, expression of ectopic FOXK2 concentrates in cell nuclei. These findings suggest that S398 mutations do not promote nuclear‐cytoplasmic shuttling of FOXK2 protein compared to the wild‐type form.

We further examined whether phosphorylation of FOXK2 at Ser398 impacts breast cancer cell growth and clonogenicity. Remarkably, cells transfected with S398A have significantly impaired growth (Figure 5C) and form fewer colonies (Figure 5D) than pFLAG‐CMV empty vector. When we followed transfected cells at different time points, we observed that metabolic cell viability is also reduced in S398A FOXK2 compared to the empty vector in 48 and 72 h (Figure 5E, Figure S10). Interestingly, metabolic cell viability was also inhibited in Ser398 phosphodeficient mutant compared with the empty vector, even in the presence of docetaxel (Figure 5F) and doxorubicin (Figure 5G) chemotherapeutic agents, an effect that was not similarly observed in S398E FOXK2. These results suggest that phosphorylation of FOXK2 at Ser 398 enhances cell viability and growth. Interestingly, higher stage breast cancer patients exhibit elevated FOXK2 phosphorylation levels at S398 (Figure S11), confirming the biological impact of these findings.

### 3.6 FOXK2 Transcription Factor Is a Substrate for AKT Phosphorylation

To explore the mechanisms underlying FOXK2 protein reduction, we treated cells transfected with FOXK2 genetic variants with the MG132 proteasome inhibitor. As shown in Figure S12A, proteasome inhibition did not result in the accumulation of the phospho‐deficient S398A form of FOXK2. To confirm the effectiveness of MG132 treatment, ubiquitinated proteins were evaluated and increased ubiquitin staining was observed in MG132‐treated cells (Figure S12B). This suggests that the reduced protein levels are unlikely to be driven by enhanced proteasomal degradation. Next, we examined whether the reduced protein levels could be explained by differences at the mRNA level. Quantitative real‐time PCR analysis revealed that cells overexpressing FOXK2 genetic variants (Figure S13A) showed similar FOXK2 mRNA levels (Figure S13B). These findings indicate that the observed differences in protein levels do not result from altered mRNA synthesis or degradation.

Next, we investigated whether FOXK2 is a target for AKT phosphorylation. For this purpose, we conducted FOXK2 immunoprecipitation on protein extracts of MDA‐MB‐231 drug‐resistant cells (Figure 6A) and stained for a phospho‐(Ser/Thr) AKT substrate (RXRXXS*/T*) antibody, which recognizes proteins containing phospho‐serine/threonine phosphorylated by AKT kinase. Figure 6B shows that staining for phospho‐AKT substrate is detected in FOXK2 eluates, further suggesting that FOXK2 might be a substrate for AKT phosphorylation. To verify the interaction between FOXK2 and AKT, we performed co‐IP experiments in MDA‐MB‐231 cells. Although detection of AKT staining in FOXK2 immunoprecipitates was not possible due to highly exposed antibody heavy chains (Figure S14), FOXK2 staining is detected as a lower mobility band in drug‐resistant cells following immunoprecipitation of phosphorylated AKT (Figure 6C,D). We have then transfected MCF‐7 cells with expression plasmid vectors encoding FOXK2 genetic variants and observed that cells overexpressing the wild‐type and S398E phosphomimic forms of FOXK2 show increased levels of phospho‐(Ser/Thr) AKT substrate staining (Figure 6E). Notably, S398A FOXK2 mutation virtually abolishes AKT substrate immunostaining (Figure 6E). Considering that serine residues can be targeted by post‐translational modifications other than phosphorylation, we have exposed protein extracts from cells overexpressing FOXK2 variants to treatment with λPP. Our data show that λPP treatment attenuates the differences in protein mobility shifts, as observed by the fast‐migrating FOXK2 bands in the gels in all lanes (Figure 6F). Remarkably, cells transfected with wild‐type FOXK2 exhibit reduced phospho‐AKT substrate staining in the presence of λPP (Figure 6F), which suggests that FOXK2, at least in part, is phosphorylated by AKT. Altogether, these results suggest that AKT might target FOXK2 for phosphorylation at Ser398.

**FIGURE 6:**
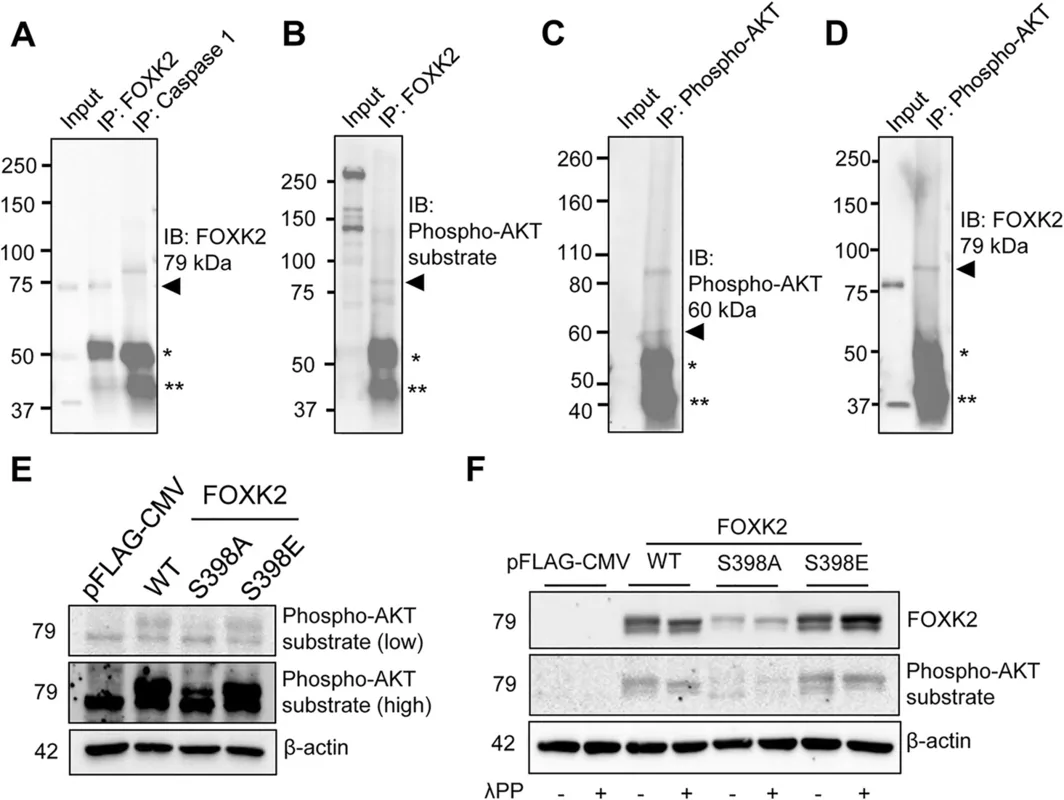
FOXK2 transcription factor is a substrate for AKT phosphorylation. (A–D) MDA‐MB‐231 cells were subjected to immunoprecipitation (IP) with anti‐FOXK2 (A and B) and Phospho‐AKT (C and D) antibodies conjugated to A/G plus agarose resins. The immunoprecipitates were analyzed by immunoblotting (IB) using the indicated antibodies. Resins conjugated to anti‐caspase‐1 antibody were used as a negative control. Single asterisks indicate 50 kDa bands that correspond to the heavy chains of the antibodies used in the protocol. Double asterisks indicate non‐specific bands. (E) Immunoblotting analyses using MCF‐7 cells transfected with the empty vector (pFLAG‐CMV), wild‐type, S398A or S398E FOXK2 variants for 24 h. (F) Immunoblotting analyses of MCF‐7 cells transfected as described in (E), with lysates treated (+) or not (−) with lambda protein phosphatase (λPP) for 1 h at 37°C.

## 4 Discussion

Breast cancer is the leading cause of cancer‐related deaths among women worldwide [47] and chemoresistance remains one of the main causes of relapse and therapeutic failure [48]. The FOXK2 transcription factor modulates drug sensitivity in breast cancer cells through the transcriptional activation of *FOXO3a*. However, chemoresistant cells exhibit high protein levels of FOXK2, which seem to be inactive [23, 29]. Despite growing evidence suggesting that FOXK2 is a target for PTMs [12, 26, 27, 28, 30], the mechanisms regulating this transcription factor in the context of breast cancer chemoresistance remain poorly understood. In this study, we investigated the role of AKT‐mediated phosphorylation in the regulation of FOXK2 in breast cancer cells.

Firstly, our results from siRNA‐based AKT inhibition support a role for AKT in regulating cell viability and chemoresistance in MDA‐MB‐231 cells. These findings are consistent with several studies reporting the involvement of AKT signaling activation in therapy resistance in breast cancer, including chemotherapy [49, 50, 51]. AKT acts as a key regulator of chemoresistance through its interaction with multiple signaling pathways and control of different cellular processes, including suppression of apoptosis and autophagy‐associated cell death, as well as positive modulation of antioxidant enzymes and membrane transporters involved in drug efflux [50]. Accordingly, PI3K/AKT inhibition represents an important therapeutic strategy, with some compounds already approved for clinical use [52, 53].

We demonstrate that both genetic and pharmacological inhibition of AKT reduce FOXK2 protein levels in chemoresistant MDA‐MB‐231 cells. Although AKT modulates FOXK2 protein levels, AKT knockdown reduced cell viability and increased sensitivity to doxorubicin regardless of ectopic FOXK2 levels in co‐transfection experiments. It is possible that these proteins are part of the same signaling pathway, in which AKT acts as an upstream regulator of FOXK2, being sufficient to modulate chemosensitivity in drug‐resistant breast cancer cells. Moreover, the AKT‐FOXK2 axis might be involved in other cellular processes such as proliferation, migration, and invasion. This is supported by growing evidence implicating both FOXK2 [12, 13, 14, 15, 16] and PI3K/AKT [54, 55, 56, 57] in the regulation of epithelial‐to‐mesenchymal transition markers and in cell migration and invasion. Also, MDA‐MB‐231 cells exhibit an intermediate phenotype between the chemosensitive MCF‐7 cells and drug‐resistant MCF‐7‐derived cell lines, in terms of drug response and FOXK2 protein levels [23]. Therefore, modulation of the AKT‐FOXK2 axis may have a more pronounced impact in other drug‐resistant models.

While previous studies have demonstrated that FOXK transcription factors are regulated by GSK3‐mediated phosphorylation in normal cellular models, such as murine fibroblasts and pre‐adipocytes [25, 45, 58], our results did not reveal changes in FOXK2 protein levels upon GSK3 inhibition. These findings suggest that the regulatory mechanisms controlling FOXK2 may be cell type‐specific. Differences in the genetic and signaling landscapes between normal and cancer cells, including alterations in upstream pathways such as KRAS signaling, may influence the contribution of GSK3 to FOXK2 regulation and account for the distinct effects observed across cellular models.

Regarding subcellular localization, our results indicate that FOXK2 is predominantly localized in the nucleus of breast cancer cells, corroborating previous reports for this transcription factor in these [23] and other cell models [24, 25, 59]. In contrast to MCF‐7 cells, phosphorylated AKT is mainly found in the nucleus of MDA‐MB‐231 cells. Interestingly, nuclear AKT [60] and AKT‐FOXO‐Bim pathway [61] have been associated with breast cancer stem cell‐like phenotype, which is extensively linked to tumor initiation and progression, metastasis and drug resistance [62]. Nuclear AKT can interact with the DNA damage repair machinery and RNA export pathways [63], as well as transcriptional factors [64].

Supporting the hypothesis that FOXK2 is regulated by phosphorylation, lambda protein phosphatase treatment resulted in a mobility shift of FOXK2 protein bands, denoting dephosphorylation and enhanced electrophoretic mobility. Consistently, FOXK2 exhibits reduced mobility in Phos‐tag gels, along with global modulation of its protein levels following AKT pathway modulation. Taken together, these data indicate that FOXK2 can be modulated by AKT‐mediated phosphorylation. Reinforcing these findings, *in silico* analyses indicate several putative phosphorylation sites in FOXK2 protein sequence, some of them potentially mediated by AKT. Also, our analyses revealed higher levels of phosphorylated FOXK2 in breast tumors compared to adjacent normal breast tissue. All these data revealed serine 398 as a potential phosphorylation site in FOXK2, the reason why we focused on the generation of phosphodeficient (S398A) and phosphomimic (S398E) FOXK2 variants. We found that, compared with the wild‐type and S398E FOXK2 forms, the S398A variant impacts cell growth, clonogenicity and cell viability in breast cancer cells, even in the presence of chemotherapeutic drugs. Although we cannot exclude the possibility that the mutation at Ser398 may influence other phosphorylation sites in FOXK2, our findings provide new insights into the mechanisms of FOXK2 regulation in breast cancer biology. Interestingly, post‐translational modifications at Ser398 of FOXK2 have not been previously described or experimentally validated, constituting a novel regulatory site in the FOXK2 puzzle.

Regarding FOXK2 protein levels, the reduction observed for the phosphodeficient S398A mutant does not appear to result from enhanced proteasomal degradation or altered mRNA levels. One possible explanation is that the loss of phosphorylation at Ser398 affects FOXK2 solubility, rendering the protein more prone to aggregation and consequently reducing its recovery during cell lysis and sample preparation. Although this possibility remains to be experimentally validated, AIUPred analysis predicts that FOXK2 contains extensive intrinsically disordered regions, including the region encompassing Ser398 [65, 66]. Such regions are particularly prone to aggregation and commonly serve as hotspots for regulatory post‐translational modifications [67].

Also, our results demonstrated that all FOXK2 genetic variants analyzed in this study remained predominantly localized in the nucleus, suggesting that mutating S398 to alanine does not affect FOXK2 subcellular localization. This finding contrasts with previous reports showing that mutations at other FOXK2 phosphorylation sites promote nucleocytoplasmic shuttling in distinct tumor models [12, 26]. Similar effects on FOXK2 intracellular distribution have also been described for mutations at SUMOylation and acetylation sites [27, 30], indicating that the functional consequences of FOXK2 post‐translational modifications may be site‐ and context‐dependent.

Notably, FOXK2 immunodetection using an antibody against AKT‐phosphorylated substrates suggests that FOXK2 is a target for AKT, with Ser398 emerging as a crucial site involved in the AKT‐FOXK2 regulatory axis. Several studies have shown that FOXK2 function and subcellular localization are regulated by phosphorylation at different sites [12, 24, 25, 26]. Interestingly, Ser398 is located close to other known phosphorylation sites on FOXK2, including serine 368 and 423 [24] and serine 415 and 419 [25]. These findings indicate that FOXK2 is tightly regulated by phosphorylation, but, to the best of our knowledge, this is the first study to report AKT as an upstream direct regulatory kinase. Nevertheless, it is worth noting that other studies have previously described indirect associations between FOXK2 and the PI3K/AKT/mTOR pathway. In cervical cancer cells, shRNA‐mediated FOXK2 knockdown reduced the levels of phosphorylated AKT and mTOR, whereas FOXK2 ectopic expression had the opposite effect [9]. Through phosphoproteomic analysis, FOXK2 has been found hypophosphorylated at serines 415 and 419 in response to insulin signaling in murine brown preadipocytes. Upon activation of the PI3K/AKT/mTOR pathway by insulin, GSK3 activity is reduced, leading to decreased phosphorylation of FOXK2 and promoting its translocation to the nucleus [25]. In addition, FOXK2 promotes clonogenicity and migration in hepatocellular carcinoma cells, at least partially through the PI3K signaling pathway. In this study, FOXK2 ectopic expression increased levels of phosphorylated AKT, survivin, c‐Myc, cyclin D1, and p27, while siRNA‐mediated FOXK2 knockdown reduced their expression [5]. This corroborates increasing evidence in the literature which points to the complexity and duality of FOXK2 functions in cancer.

In summary, our results demonstrate that FOXK2 is regulated by AKT‐mediated phosphorylation in drug‐resistant breast cancer cells. Given that FOXK2 has been increasingly reported to undergo PTMs in different cell models, including SUMOylation in breast cancer, we cannot exclude the contribution of additional PTMs in drug resistance. Notably, Ser398 emerges as a previously unexplored crucial regulatory site in FOXK2, with impacts on metabolic cell viability and clonogenicity in breast cancer cells. Considering the novelty of these findings in the context of FOXK2 regulation, further characterization of these genetic variants is warranted.

## Author Contributions

**L. T. Carneiro:** writing – original draft, conceptualization, methodology, investigation, formal analysis, data curation. **J. M. R. C. Nunes:** writing – review and editing, methodology, investigation, formal analysis. **G. De Gregoriis:** writing – review and editing, methodology, investigation, formal analysis. **B. S. Mendonça:** writing – review and editing, methodology, investigation, formal analysis. **W. M. Araújo:** writing – review and editing, methodology, formal analysis. **R. C. Maia:** writing – review and editing, resources. **E. W. F. Lam:** writing – review and editing, conceptualization, methodology, investigation. **R. S. Carvalho:** writing – review and editing, methodology, investigation, formal analysis, resources. **M. A. Carvalho:** writing – review and editing, methodology, investigation, formal analysis, resources. **A. D. Sharrocks:** writing – review and editing, methodology, investigation, formal analysis. **G. Nestal de Moraes:** writing – original draft, conceptualization, investigation, formal analysis, data curation, supervision, project administration, resources, funding acquisition.

## Funding

This work was supported by grants from L'óreal‐UNESCO‐ABC For women in science, Carlos Chagas Filho Foundation for Research Support of the State of Rio de Janeiro (FAPERJ grant numbers SEI‐26003/004812/2021, SEI‐260003/001554/2022, SEI‐260003/013154/2024 and 260003/007036/2022) and the National Council for Scientific and Technological Development (CNPq Grants 307427/2022‐6 and 408314/2021‐4 and INCT program, Grant 408340/2024‐0). L.T.C and J.M.R.C.N are supported by CNPq fellowships. R.C.M is supported by FAPERJ. The funding agencies have not been involved in the study design, data collection, analysis and interpretation or writing of the manuscript.

## Conflicts of Interest

The authors declare no conflicts of interest.

## Supporting information


**Figure S1:** FOXK2 band profile is not modulated by GSK3β knockdown. (A) Schematic representation of the experimental design. (B and C) Immunoblotting analyses using MDA‐MB‐231 cells transfected with GSK3β siRNA or exposed to the “Mock” condition (all transfection reagents except RNAs) for 24 (B) and 48 h (C).
**Figure S2:** AKT pharmacological inhibition by MK‐2206 induces MDA‐MB‐231 cell sensitivity. (A and B) MTT assays were carried out using MDA‐MB‐231 cells treated with increasing concentrations of MK‐2206 for the indicated time points. Statistical significance was determined by Two‐WOay ANOVA, comparing the optical densities (ODs) means to the mean at 0 h for each treatment condition (A) and the normalized means to the DMSO mean for each treatment time point (B) (*p ≤ 0.05; **p ≤ 0.01; ***p ≤ 0.001; ****p ≤ 0.0001; significant).
**Figure S3:** AKT pharmacological inhibition following treatment with MK‐2206. Immunoblotting analyses using MDA‐MB‐231 cells treated with DMSO vehicle control or 25 μM MK‐2206 for 4, 8, 16 and 24 h. The intensities of the unsaturated bands were determined using ImageJ. Densitometric ratios are shown below the Western blot bands.
**Figure S4:** FOXK2 and phosphorylated AKT are located in the nucleus of MDA‐MB‐231 cells. Immunoblotting analyses of cytoplasmic and nuclear fractions from MCF‐7 and MDA‐MB‐231 cells, using the NE‐PER fractioning kit (Thermo Scientific). Lamin B levels were used as a nuclear loading control, while GAPDH levels were used as a cytoplasmic loading control. C: Cytoplasm; N:Nucleus.
**Figure S5:** AKT modulates cell growth and response to chemotherapy, regardless of FOXK2 levels. (A) Cell count with trypan blue exclusion using MDA‐MB‐231 cells transfected with non‐silencing control (NSC) or AKT1 siRNA for 48 h and transfected with the empty vector (pCMV5) or wild‐type FOXK2 for 24 h. (B) MTT assays were carried out using transfected cells as described in (A) treated with increasing concentrations of doxorubicin for 72 h. Statistical significance was determined by One‐Way or Two‐Way ANOVA (ns, not significant; *p ≤ 0.05; **p ≤ 0.01; ***p ≤ 0.001; ****p ≤ 0.0001, significant).
**Figure S6:** AKT inhibition results in changes in TFOXK2 protein mobility in Phos‐tag gels. (A) Immunoblotting analyses on Phos‐tagTM SDS‐PAGE polyacrylamide gel using MDA‐MB‐231 cells treated with 25 μM MK‐2206 for 24 h. (B and C) Immunoblotting analyses on Phos‐tagTM SDS‐PAGE polyacrylamide gel using MDA‐MB‐231 cells transfected with non‐silencing control (NSC) or AKT1 siRNA for 72 h (B) or transfected with NSC or AKT1 siRNA for 48 h and co‐transfected with the empty (pCMV5) or the wild‐type FOXK2 vector for 24 h (C).
**Figure S7:** FOXK2 protein levels are not induced by 4‐hydroxytamoxifen treatment alone. As experimental controls, MCF‐7 and MDA‐MB‐231 cells untransfected with pBABE‐(myr)Akt:ER vector (myrAKT) and treated with 200 nM 4‐hydroxytamoxifen (4‐OHT) or ethanol vehicle control for 24 h were analyzed by immunoblotting. FOXK2 protein levels were seemingly assessed, with Hsc70 used as a constitutive control.
**Figure S8:** Overexpression of constitutively active AKT results in changes in FOXK2 protein mobility in Phos‐tag gels. FOXK2 immunoblotting analyses on Phos‐tagTM SDS‐PAGE polyacrylamide gel using MCF‐7 cells transfected with pBABE‐(myr)Akt:ER vector and treated with 200 nM 4 hydroxytamoxifen (4‐OHT) or ethanol vehicle control for 24 h.
**Figure S9:** FOXK2 subcellular localization in cells overexpressing FOXK2 genetic variants mutated for serine 398. MCF‐7 cells were transfected with the empty vector (pFLAG‐CMV), wild‐type, S398A or S398E FOXK2 variants for 24 h and had their cytoplasmic and nuclear fractions separated by the NE PER kit (A). Subsequently, fractioned (A) lysates were subjected to the analysis of FOXK2 protein levels by Western blotting. Lamin B was used as a nuclear constitutive control, while GAPDH as cytoplasmic constitutive control. (B) MCF‐7 were seeded into slides, transfected for 24 h and submitted to the immunofluorescence protocol using anti‐FLAG staining. Subcellular localization of FOXK2 was analyzed by confocal microscopy and nuclei were counterstained with DAPI. Scale bar: 50 μM.
**Figure S10:** FOXK2 mutation at Serine398 impairs breast cancer viability. (A and B) MTT assays were performed using MCF‐7 cells transfected with the empty vector (pFLAG‐CMV), wild‐type, S398A or S398E FOXK2 variants for 24 h, followed by treatment with increasing concentrations of docetaxel (A) or doxorubicin (B) for 48 h. Statistical significance was determined by Two‐Way ANOVA (*p ≤ 0.05, significant).
**Figure S11:** Advanced stage breast cancer samples exhibit higher levels of phosphorylated FOXK2 at S398. Analysis of phosphorylated FOXK2 levels at serine 398 (S398) in breast cancer tumor samples with different stages and normal tissue samples adjacent to the tumor (*n* = 18), using data from the Clinical Proteomic Tumor Analysis Consortium (CPTAC), available at UALCAN (http://ualcan.path.uab.edu). Statistical significance was determined by the Pearson's correlation between the log2‐transformed gene‐level molecular values and the stage as translated into a numerical value (*p ≤ 0.05; **p ≤ 0.01; ***p ≤ 0.001, significant).
**Figure S12:** The lower protein levels of the S398A form of FOXK2 are not due to proteasome degradation. A and B Immunoblotting analyses using MCF 7 cells transfected with the empty vector (pFLAG‐CMV), wild‐type, S398A or S398E FOXK2 variants for 24 h, treated or not with MG132, prior to cell lysis. The intensities of the unsaturated bands were determined using ImageJ. Densitometric ratios of FOXK2/β actin constitutive control (normalized to 1) are shown below the western blot bands.
**Figure S13:** The lower protein content of the S398A variant of FOXK2 does not result from variations in mRNA levels compared to other variants. A Immunoblotting analyses using MCF‐7 cells transfected with the empty vector (pFLAG CMV), wild‐type, S398A or S398E FOXK2 variants for 24 h. B Quantification of FOXK2 mRNA levels by real‐time quantitative PCR (SYBR Green Master Mix method) was carried out using transfected cells as described in (A). Statistical significance was determined by one‐way ANOVA (ns, not significant).
**Figure S14:** Phosphorylated AKT staining following FOXK2 immunoprecipitation in MDA‐MB‐231 cells. (A and B) MDA‐MB‐231 cells were subjected to immunoprecipitation (IP) with anti‐FOXK2 antibody conjugated to A/G plus agarose resins. The immunoprecipitates were analyzed by immunoblotting (IB) using FOXK2 (A) and Phospho‐AKT (B) antibodies. The resins conjugated to anti caspase1 antibody were used as a negative control and the inputs were reserved after the pre‐clear step. Single asterisks indicate 50 kDa bands that correspond to the heavy chains of the immunoglobulins used in the protocol. Double asterisks indicate non‐specific bands.

**Table S1:** Primers used for mutagenesis by overlap extension.

**Table S2:** Primers used for sequencing of FOXK2 variants.

## Data Availability

Most of the results generated during this study are included in this article. Additional data can be made available upon request.
