# Supplementary material for: AKT Phosphorylation of FOXK2 at Ser398 Impacts Breast Cancer Cell Growth and Viability
Source: FASEB J. 2026 Oct 13;40(20):e72373. doi: 10.1096/fj.202600413RRR (PMC13660766; doi:10.1096/fj.202600413RRR)
Supplement: Supplementary file 1 [file FSB2-40-e72373-s001.pdf]

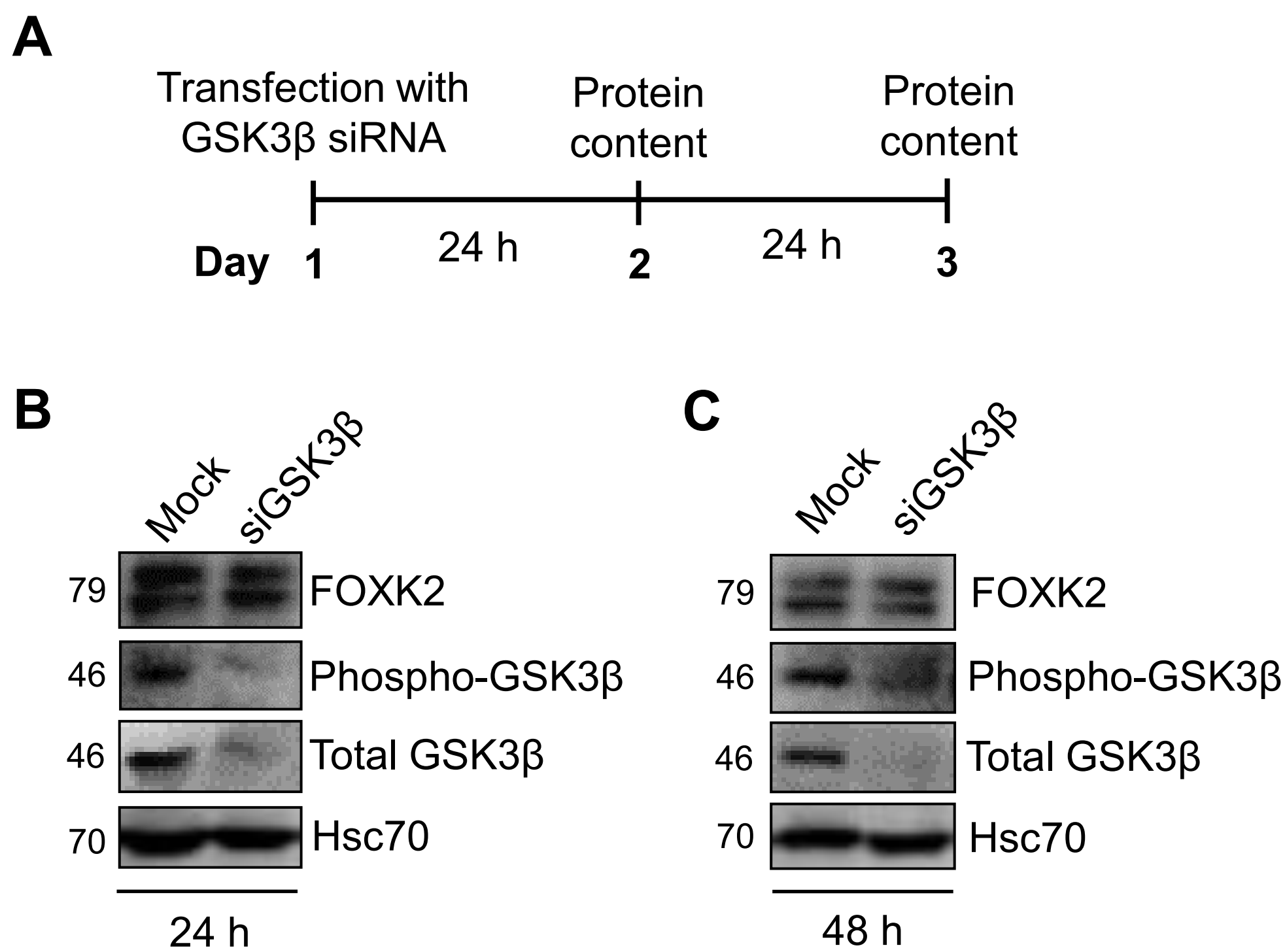


**Supplementary Fig. 1 – FOXK2 band profile is not modulated by GSK3β knockdown**. (**A**) Schematic representation of the experimental design. (**B** and **C**) Immunoblotting analyses using MDA-MB-231 cells transfected with GSK3β siRNA or exposed to the 'Mock' condition (all transfection reagents except RNAs) for 24 (B) and 48 h (C).

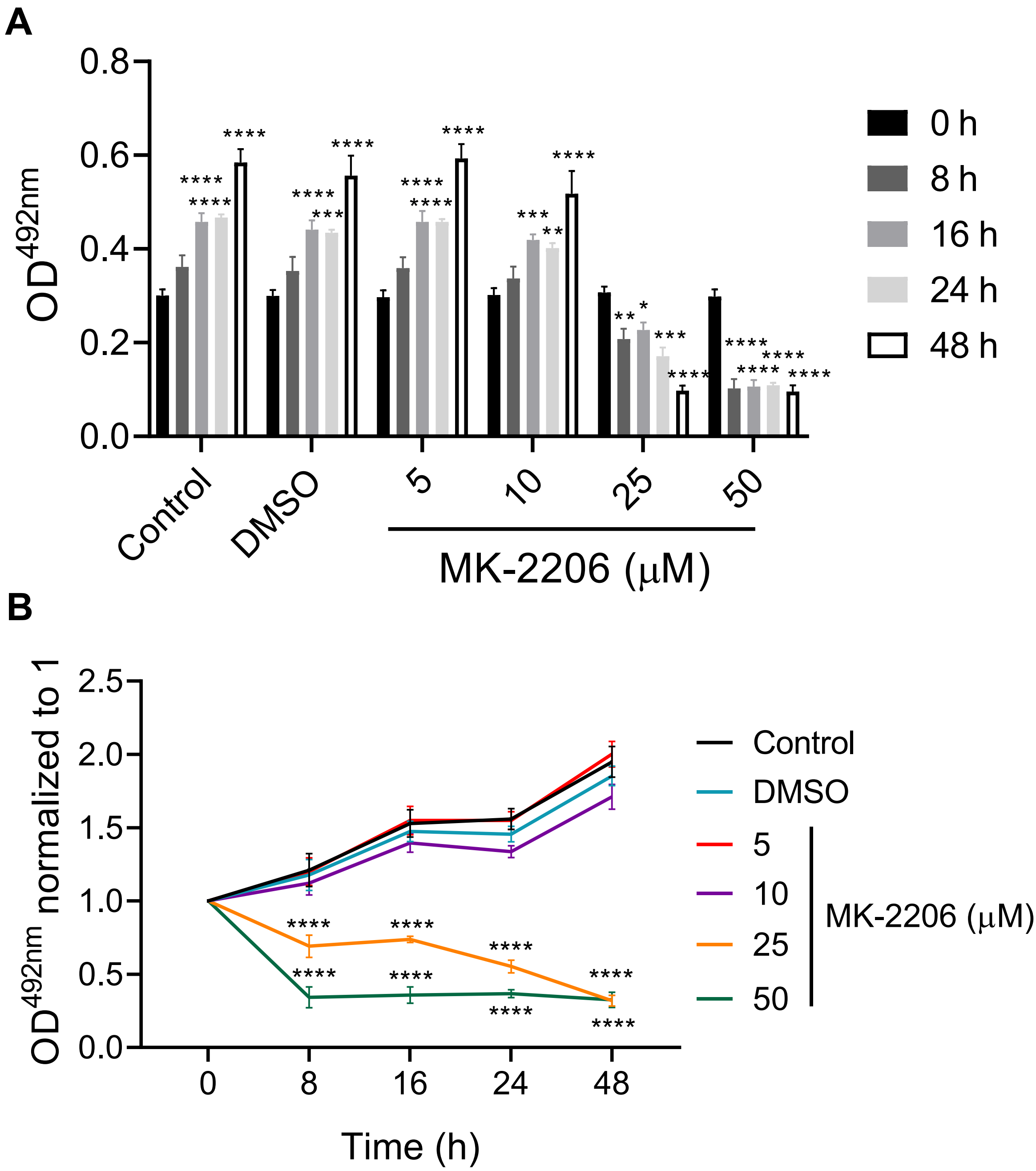


**Supplementary Fig. 2 - AKT pharmacological inhibition by MK-2206 induces MDA-MB-231 cell sensitivity**. (**A** and **B**) MTT assays were carried out using MDA-MB-231 cells treated with increasing concentrations of MK-2206 for the indicated time points. Statistical significance was determined by two-way ANOVA, comparing the optical densities (ODs) means to the mean at 0 h for each treatment condition (A) and the normalized means to the DMSO mean for each treatment time point (B) (*$p \leq 0,05$; ** $p \leq 0,01$; *** $p \leq 0,001$; **** $p \leq 0,0001$; significant).

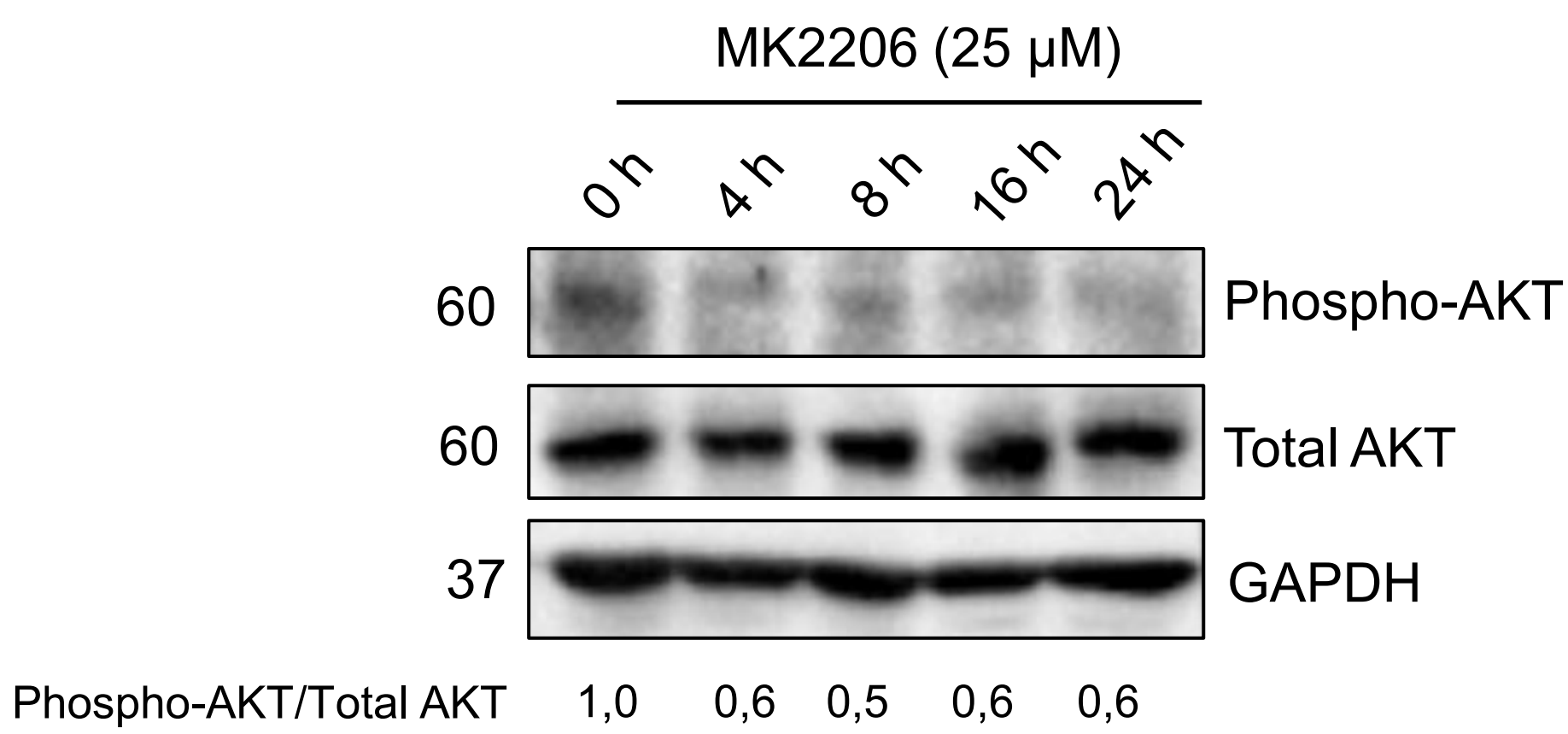


**Supplementary Fig. 3 – AKT pharmacological inhibition following treatment with MK-2206.** Immunoblotting analyses using MDA-MB-231 cells treated with DMSO vehicle control or 25 µM MK-2206 for 4, 8, 16 and 24 h. The intensities of the unsaturated bands were determined using ImageJ. Densitometric ratios are shown below the Western blot bands.

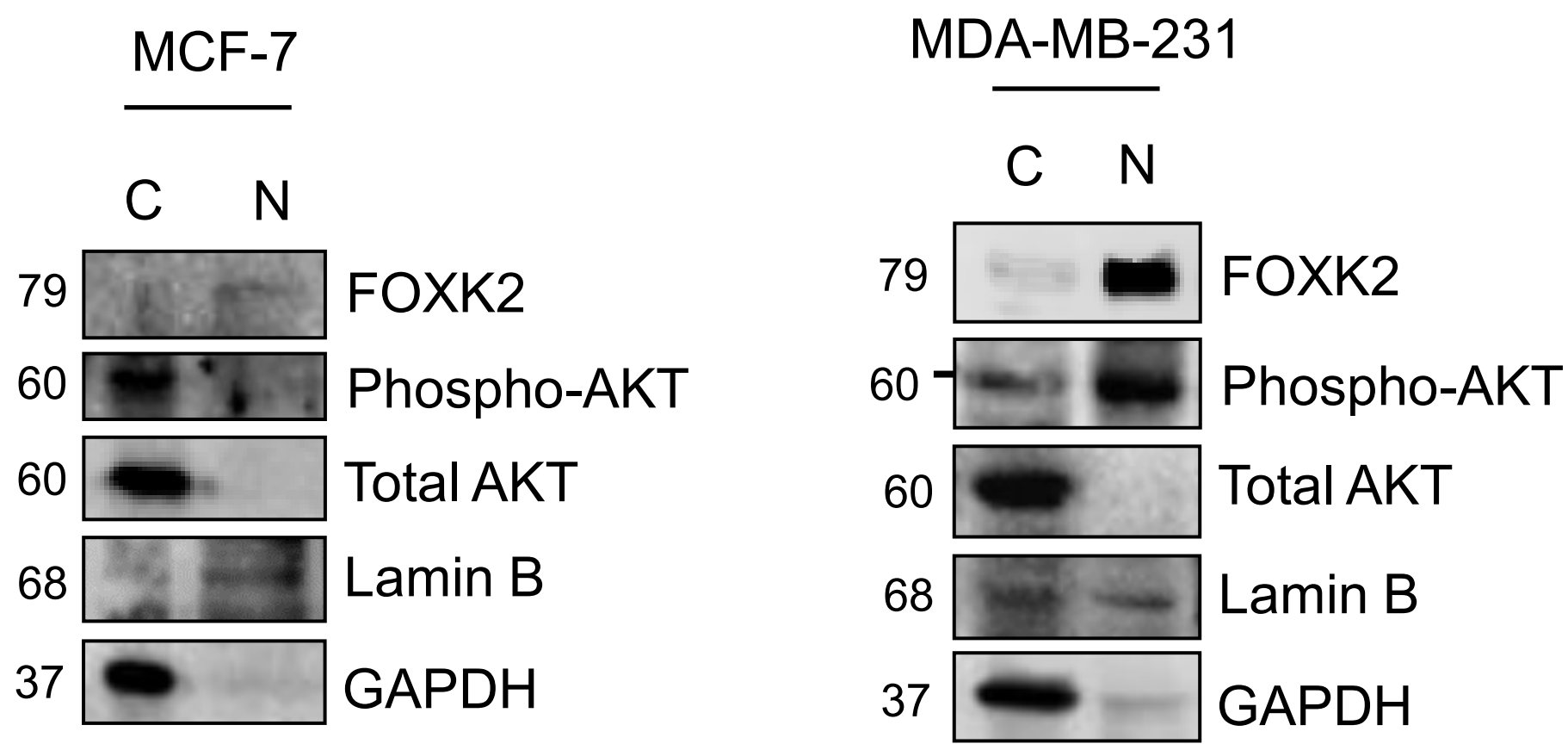


**Supplementary Fig. 4 – FOXK2 and phosphorylated AKT are located in the nucleus of MDA-MB-231 cells**. Immunoblotting analyses of cytoplasmic and nuclear fractions from MCF-7 and MDA-MB-231 cells, using the NE-PER fractioning kit (Thermo Scientific). Lamin B levels were used as a nuclear loading control, while GAPDH levels were used as a cytoplasmic loading control. C: Cytoplasm; N:Nucleus.

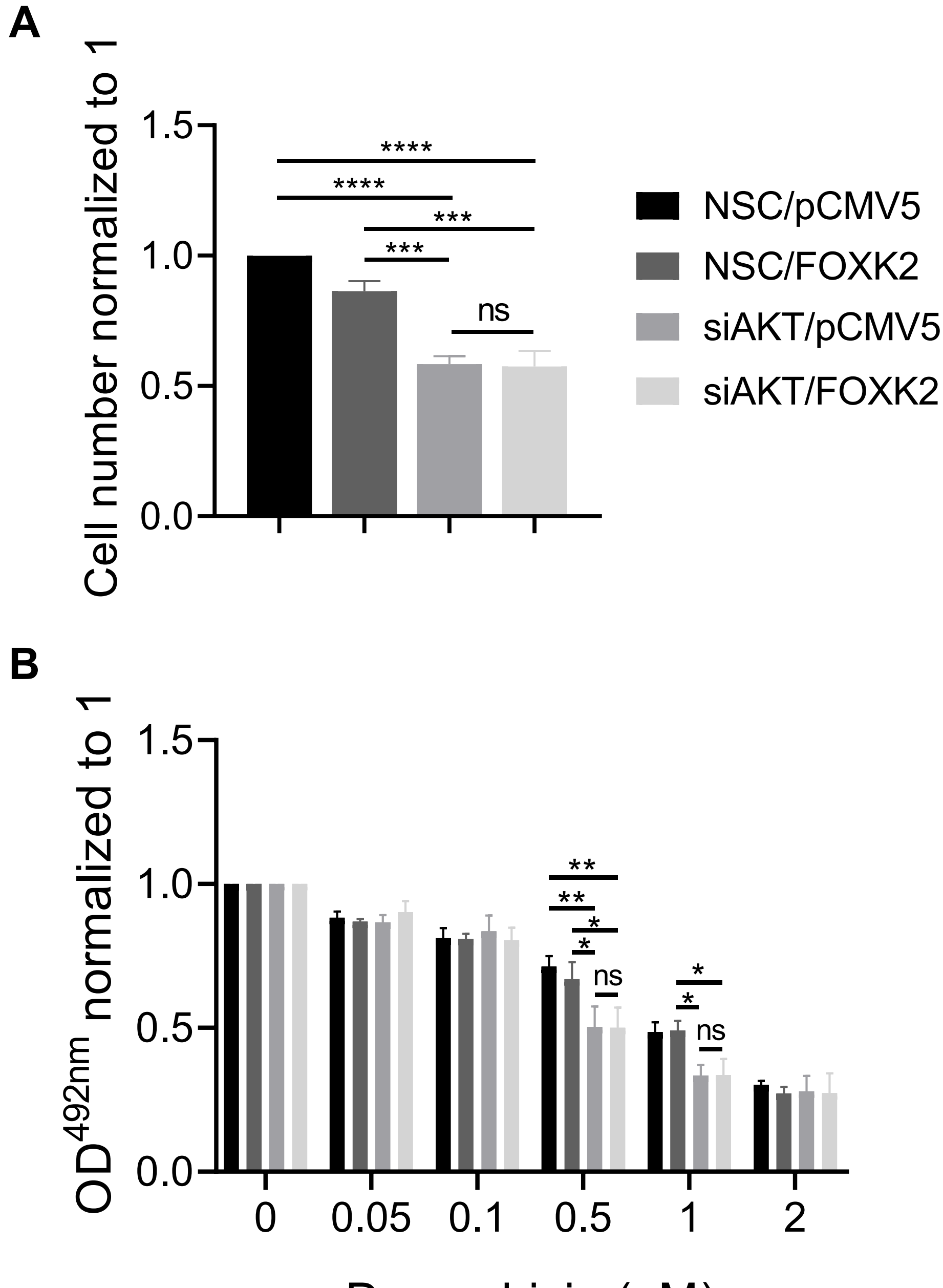


**Supplementary Fig. 5 – AKT modulates cell growth and response to chemotherapy, regardless of FOXK2 levels**. (**A**) Cell count with trypan blue exclusion using MDA-MB-231 cells transfected with non-silencing control (NSC) or AKT1 siRNA for 48 h and transfected with the empty vector (pCMV5) or wild-type FOXK2 for 24 h. (**B**) MTT assays were carried out using transfected cells as described in (A) treated with increasing concentrations of doxorubicin for 72 h. Statistical significance was determined by one-way or two-way ANOVA (ns, not significant; * $p \leq 0,05$; ** $p \leq 0,01$; *** $p \leq 0,001$; **** $p \leq 0,0001$, significant).

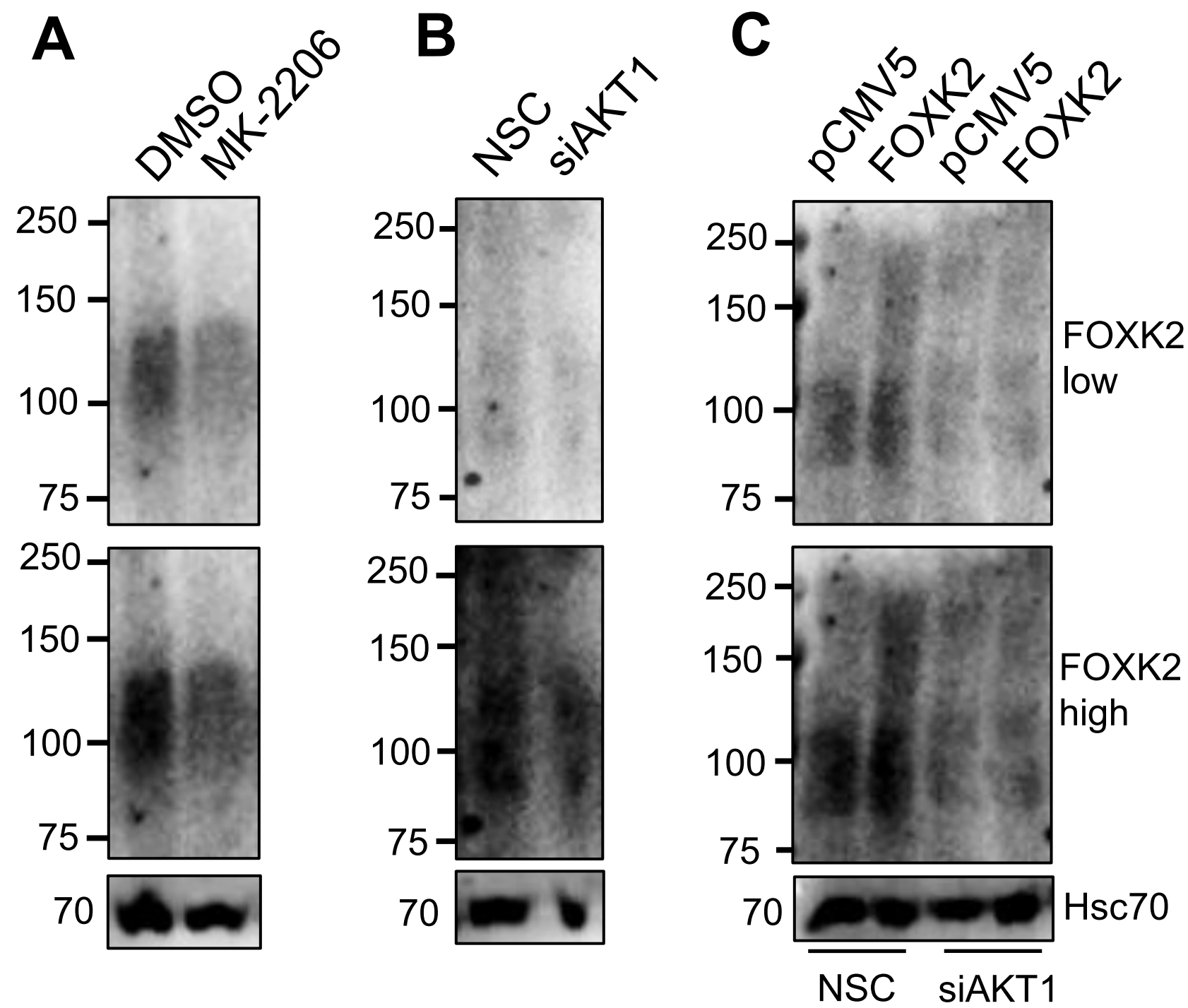


**Supplementary Fig. 6 – AKT inhibition results in changes in FOXK2 protein mobility in Phos-tag gels. (A)** Immunoblotting analyses on Phos-tag$^{TM}$ SDS-PAGE polyacrylamide gel using MDA-MB-231 cells treated with 25 μM MK-2206 for 24 h. (**B** and **C**) Immunoblotting analyses on Phos-tag$^{TM}$ SDS-PAGE polyacrylamide gel using MDA-MB-231 cells transfected with non-silencing control (NSC) or AKT1 siRNA for 72 h (B) or transfected with NSC or AKT1 siRNA for 48 h and co-transfected with the empty (pCMV5) or the wild-type FOXK2 vector for 24 h (C).

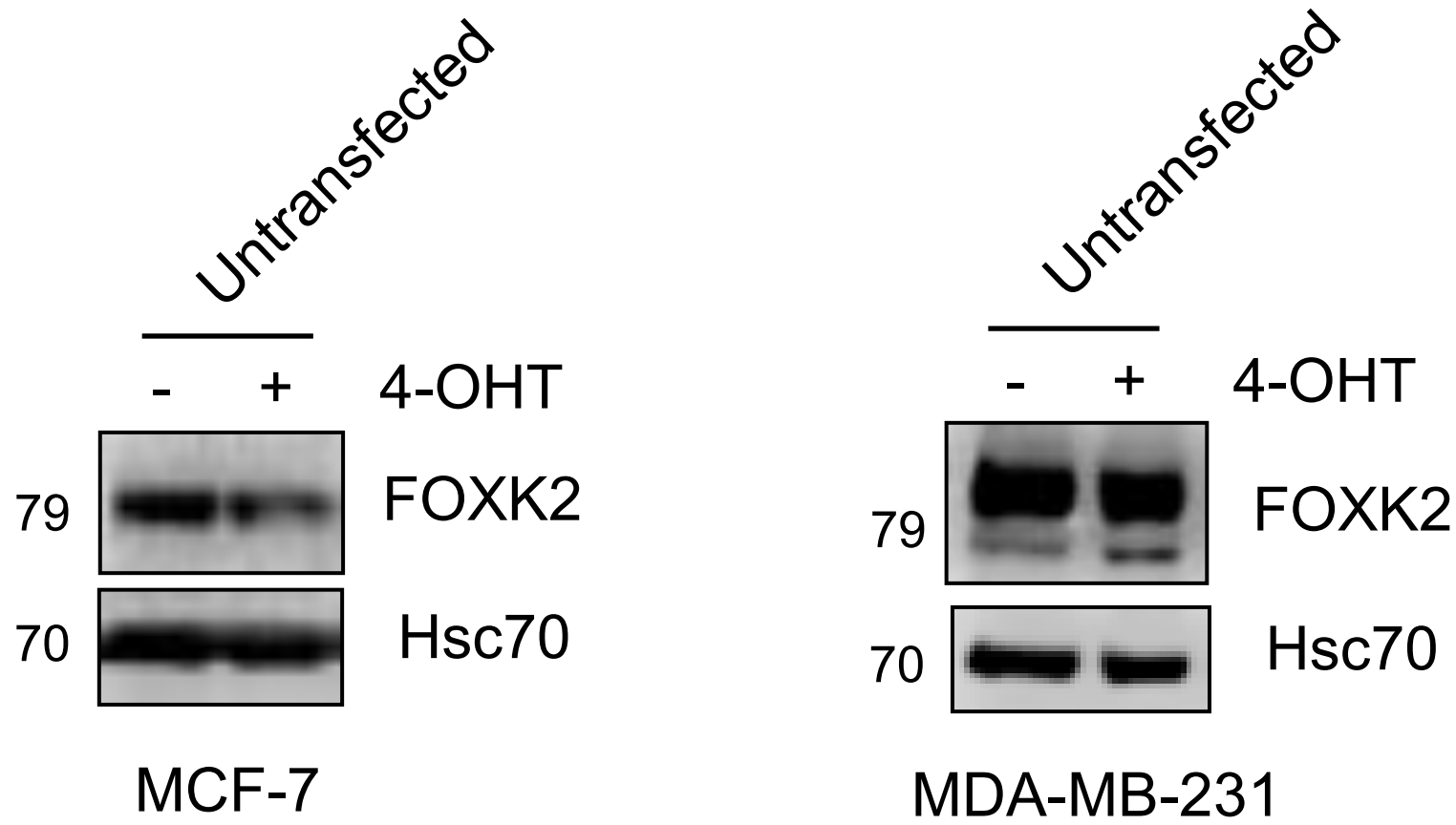


**Supplementary Fig. 7 – FOXK2 protein levels are not induced by 4-hydroxytamoxifen treatment alone**. As experimental controls, MCF-7 and MDA-MB-231 cells untransfected with pBABE-(myr)Akt:ER vector (myrAKT) and treated with 200 nM 4-hydroxytamoxifen (4-OHT) or ethanol vehicle control for 24 h were analysed by immunoblotting. FOXK2 protein levels were seemingly assessed, with Hsc70 used as a constitutive control.

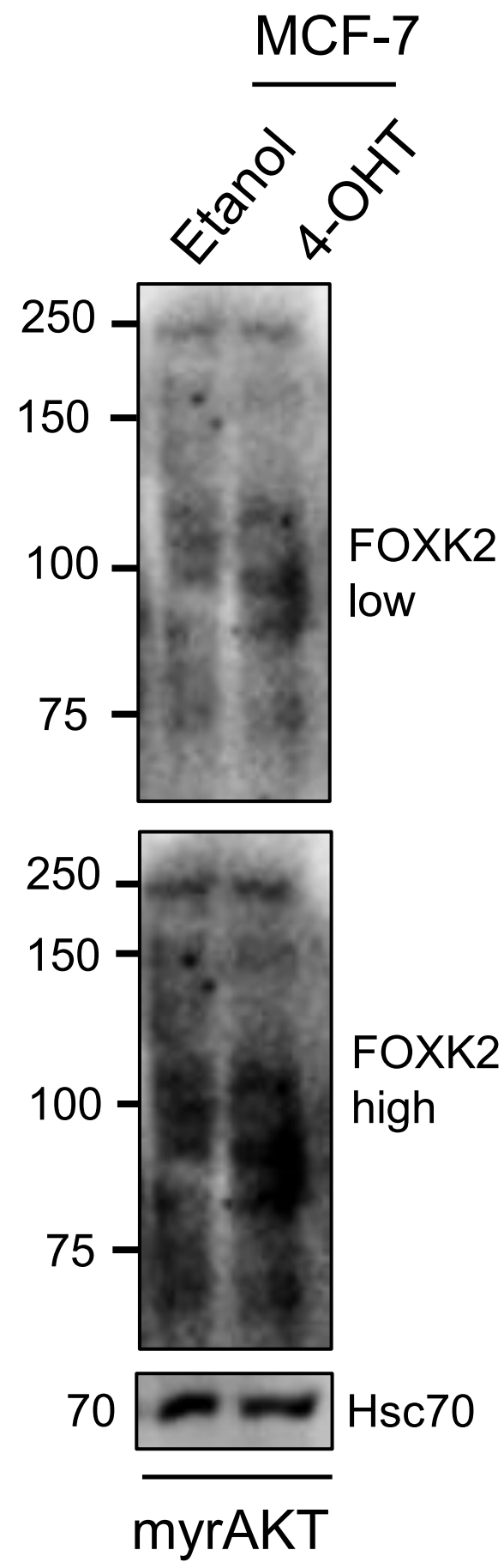


**Supplementary Fig. 8 – Overexpression of constitutively active AKT results in changes in FOXK2 protein mobility in Phos-tag gels.** FOXK2 immunoblotting analyses on Phos-tag$^{TM}$ SDS-PAGE polyacrylamide gel using MCF-7 cells transfected with pBABE-(myr)Akt:ER vector and treated with 200 nM 4-hydroxytamoxifen (4-OHT) or ethanol vehicle control for 24 h.

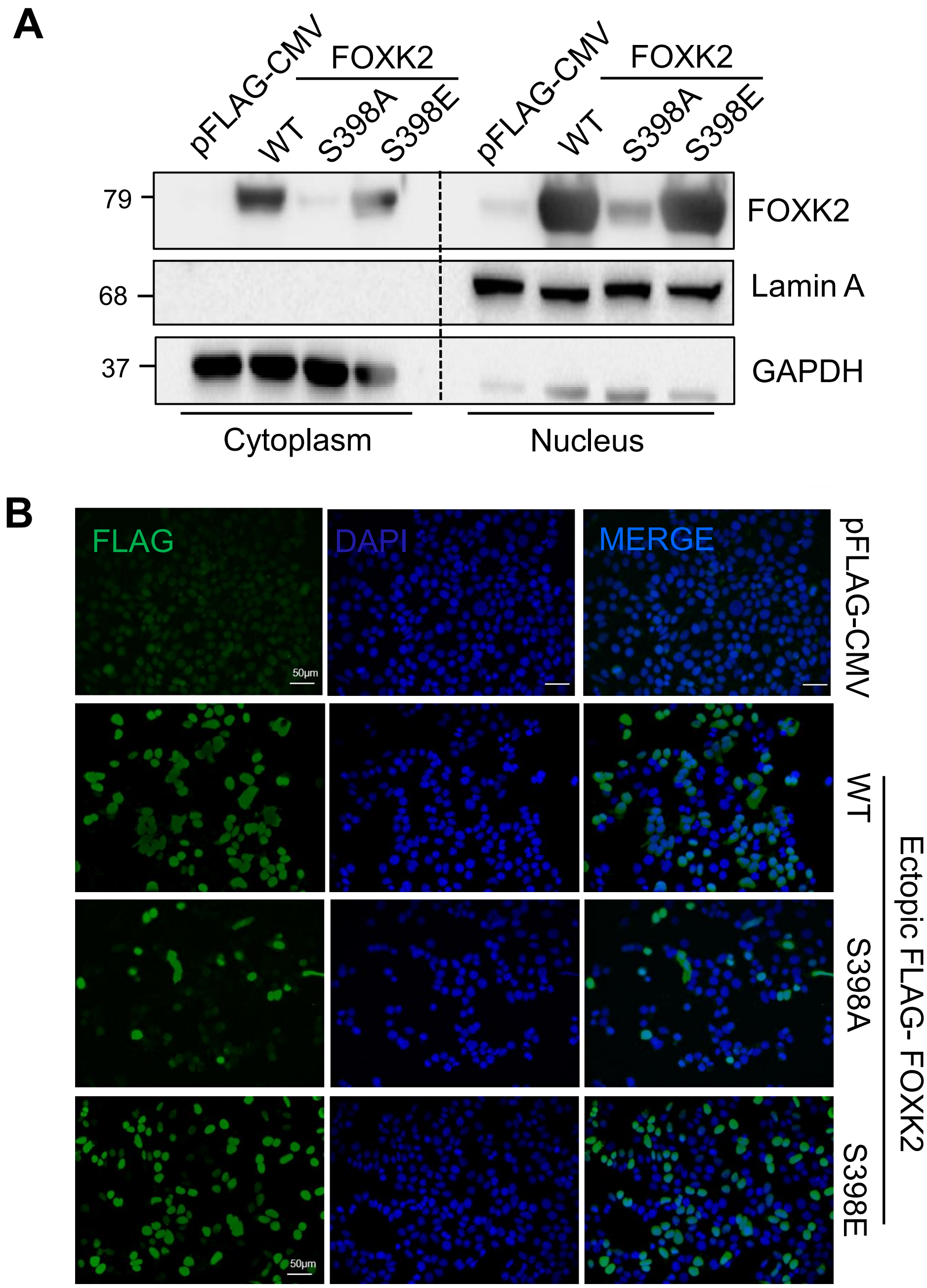


**Supplementary Figure 9 – FOXK2 subcellular localization in cells overexpressing FOXK2 genetic variants mutated for serine 398.** MCF-7 cells were transfected with the empty vector (pFLAG-CMV), wild-type, S398A or S398E FOXK2 variants for 24 h and had their cytoplasmic and nuclear fractions separated by the NE-PER kit (**A**). Subsequently, fractioned (**A**) lysates were subjected to the analysis of FOXK2 protein levels by Western blotting. Lamin B was used as a nuclear constitutive control, while GAPDH as cytoplasmic constitutive control. (**B**) MCF-7 were seeded into slides, transfected for 24 h and submitted to the immunofluorescence protocol using anti-FLAG staining. Subcellular localization of FOXK2 was analyzed by confocal microscopy and nuclei were counterstained with DAPI. Scale bar: 50 μM.

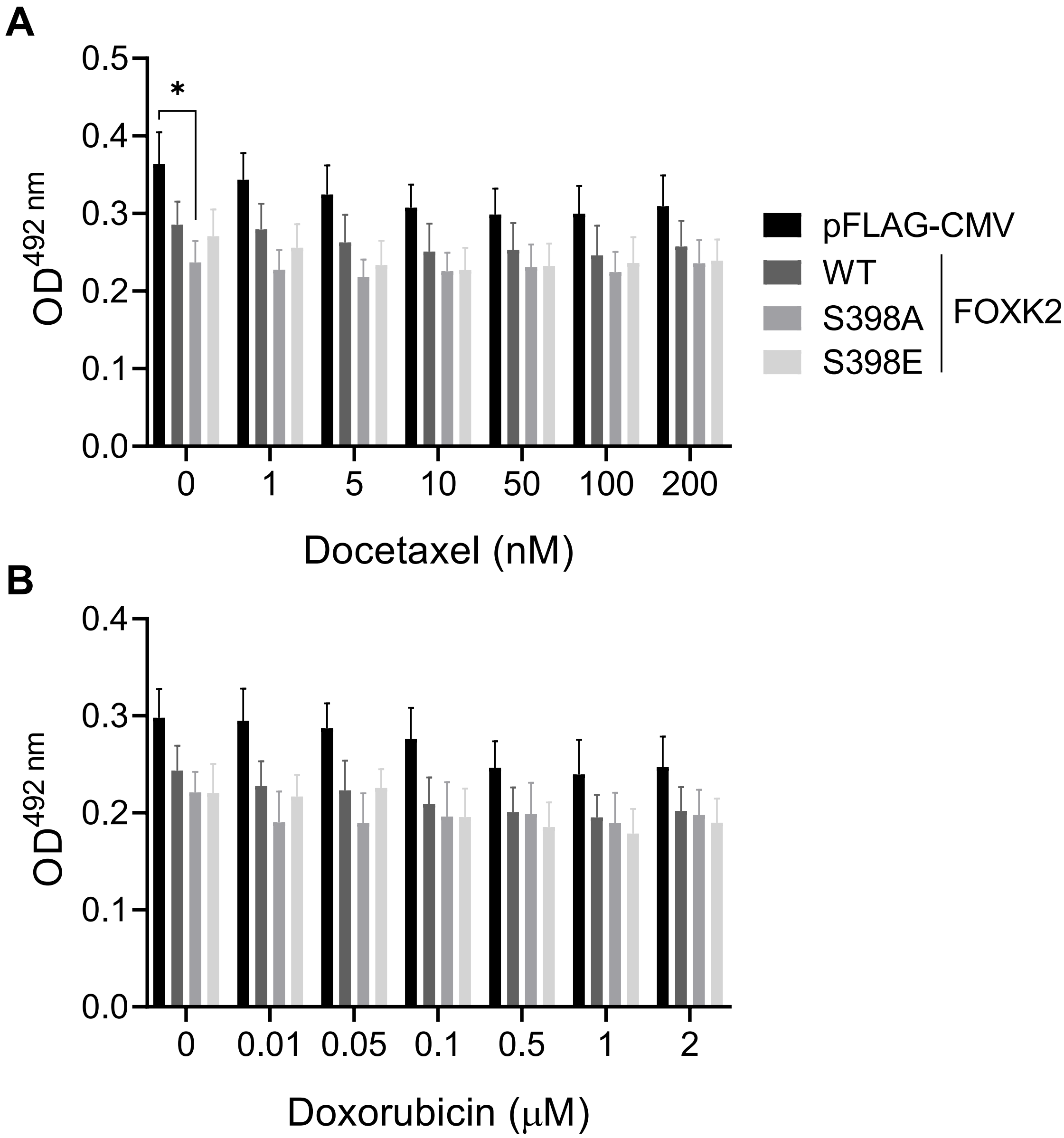


**Supplementary Fig. 10 – FOXK2 mutation at Serine398 impairs breast cancer viability.** (**A** and **B**) MTT assays were performed using MCF-7 cells transfected with the empty vector (pFLAG-CMV), wild-type, S398A or S398E FOXK2 variants for 24 hours, followed by treatment with increasing concentrations of docetaxel (A) or doxorubicin (B) for 48 hours. Statistical significance was determined by two-way ANOVA (* $p \leq 0{,}05$, significant).

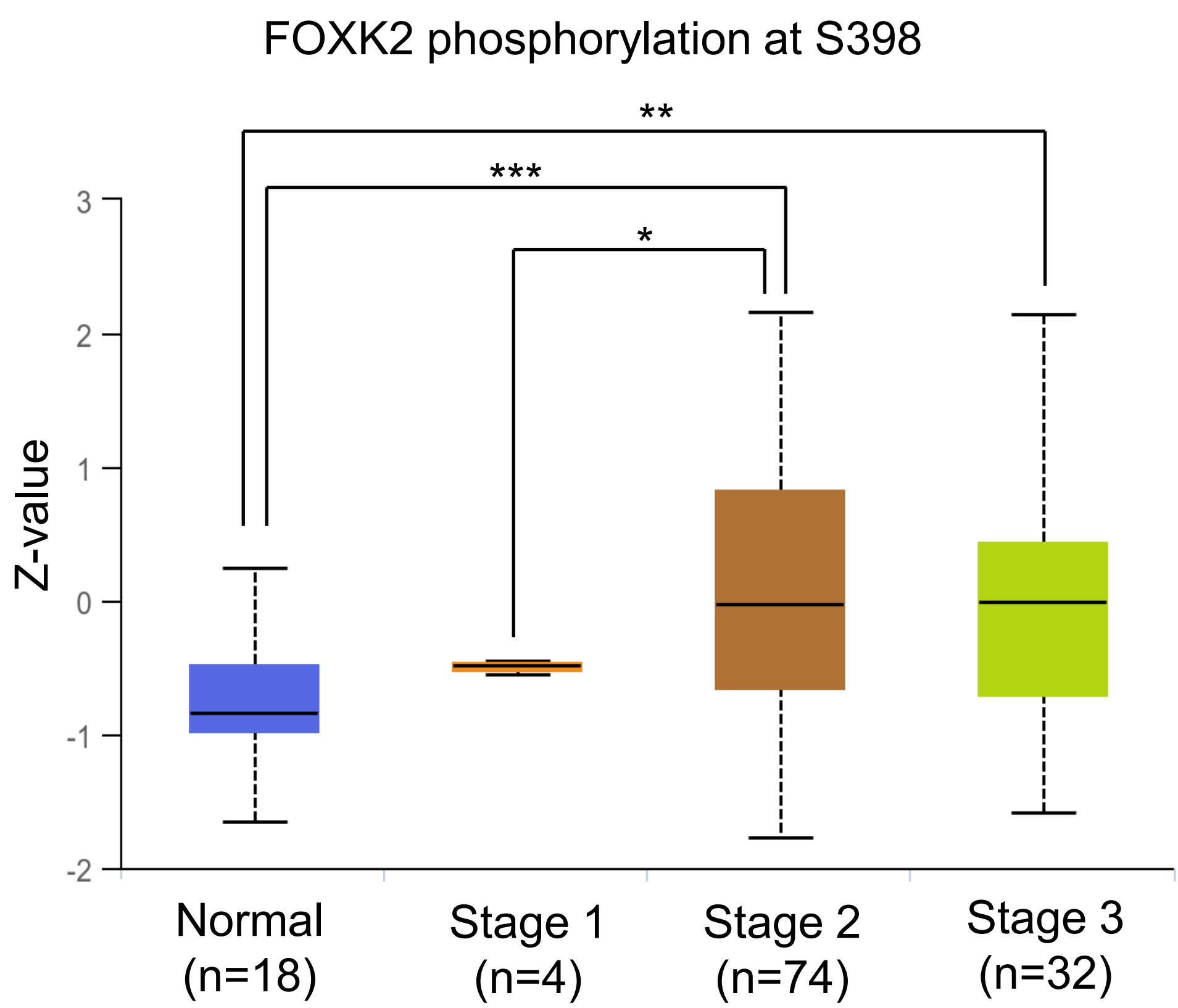


**Supplementary Fig. 11 – Advanced stage breast cancer samples exhibit higher levels of phosphorylated FOXK2 at S398.** Analysis of phosphorylated FOXK2 levels at serine 398 (S398) in breast cancer tumor samples with different stages and normal tissue samples adjacent to the tumor (n=18), using data from the Clinical Proteomic Tumor Analysis Consortium (CPTAC), available at UALCAN (http://ualcan.path.uab.edu). Statistical significance was determined by the Pearson's correlation between the log2-transformed gene-level molecular values and the stage as translated into a numerical value (* $p \leq 0.05$; ** $p \leq 0.01$; *** $p \leq 0.001$, significant).

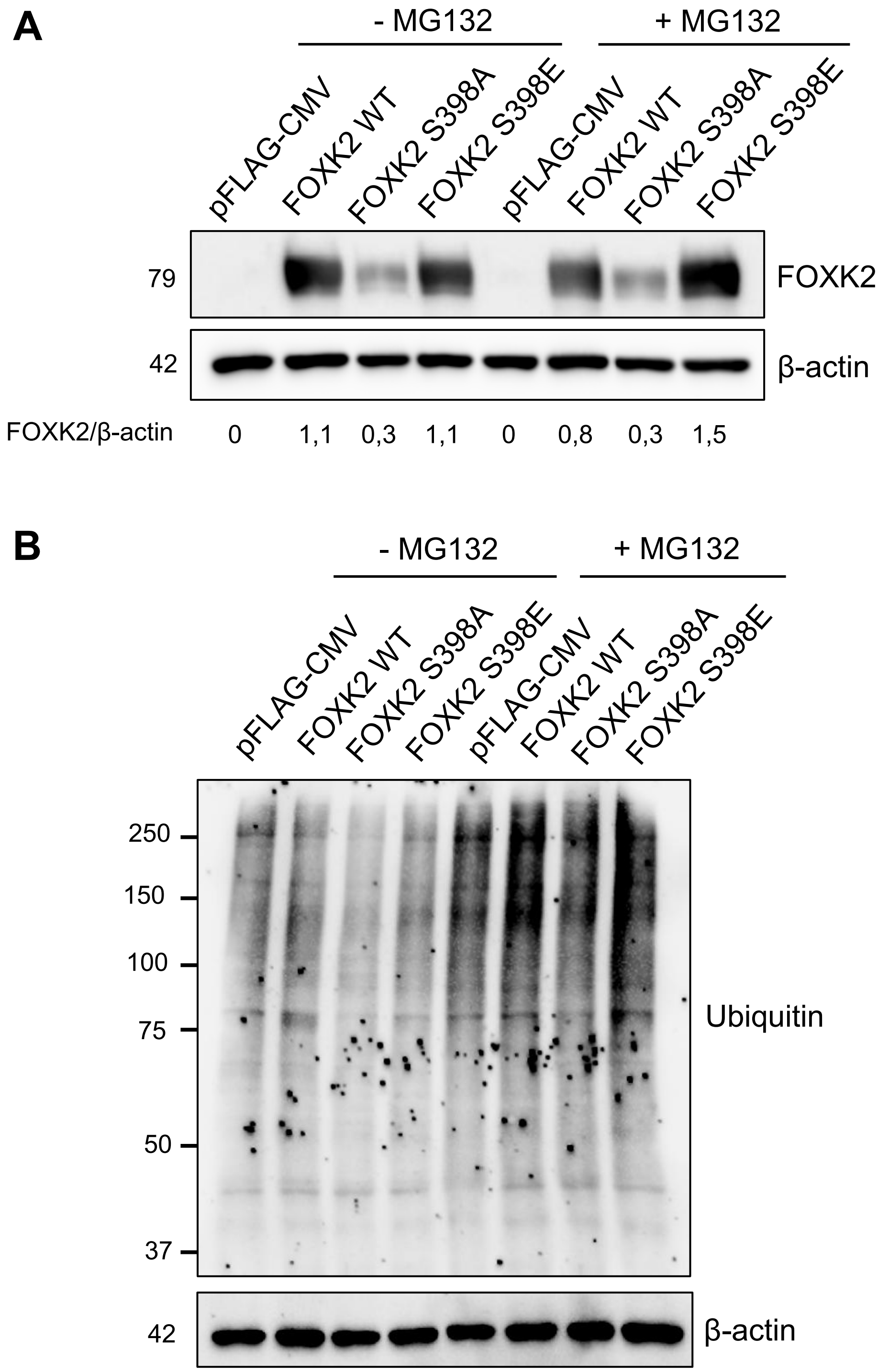


**Supplementary Figure 12 – The lower protein levels of the S398A form of FOXK2 are not due to proteasome degradation. A** and **B** Immunoblotting analyses using MCF-7 cells transfected with the empty vector (pFLAG-CMV), wild-type, S398A or S398E FOXK2 variants for 24 h, treated or not with MG132, prior to cell lysis. The intensities of the unsaturated bands were determined using ImageJ. Densitometric ratios of FOXK2/β-actin constitutive control (normalized to 1) are shown below the Western blot bands.

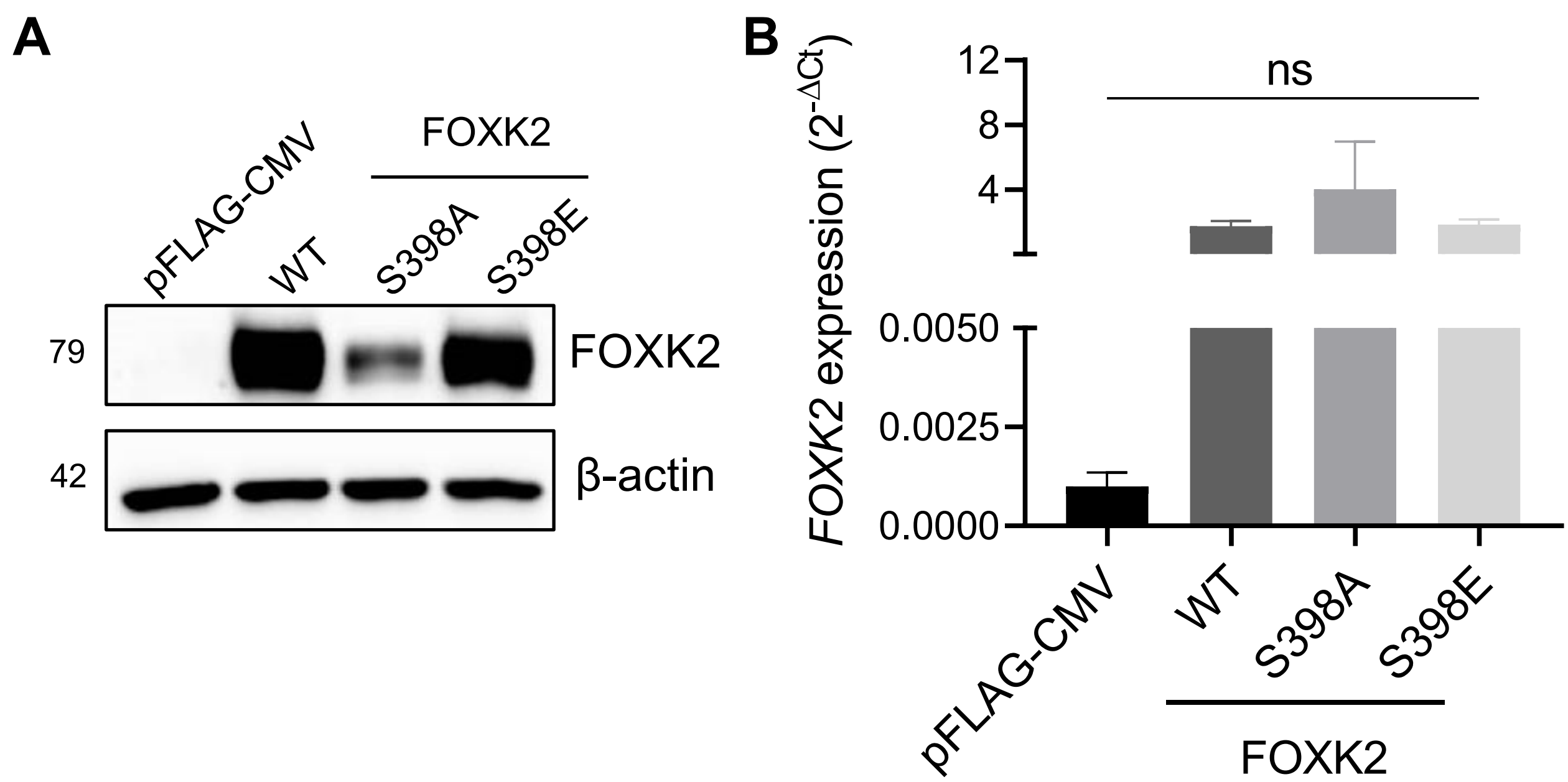


**Supplementary Fig. 13 – The lower protein content of the S398A variant of FOXK2 does not result from variations in mRNA levels compared to other variants**. **A** Immunoblotting analyses using MCF-7 cells transfected with the empty vector (pFLAG-CMV), wild-type, S398A or S398E FOXK2 variants for 24 h. **B** Quantification of RNA levels by real-time quantitative PCR (SYBR Green Master Mix method) was carried out using transfected cells as described in (A). Statistical significance was determined by one-way ANOVA (ns, not significant).

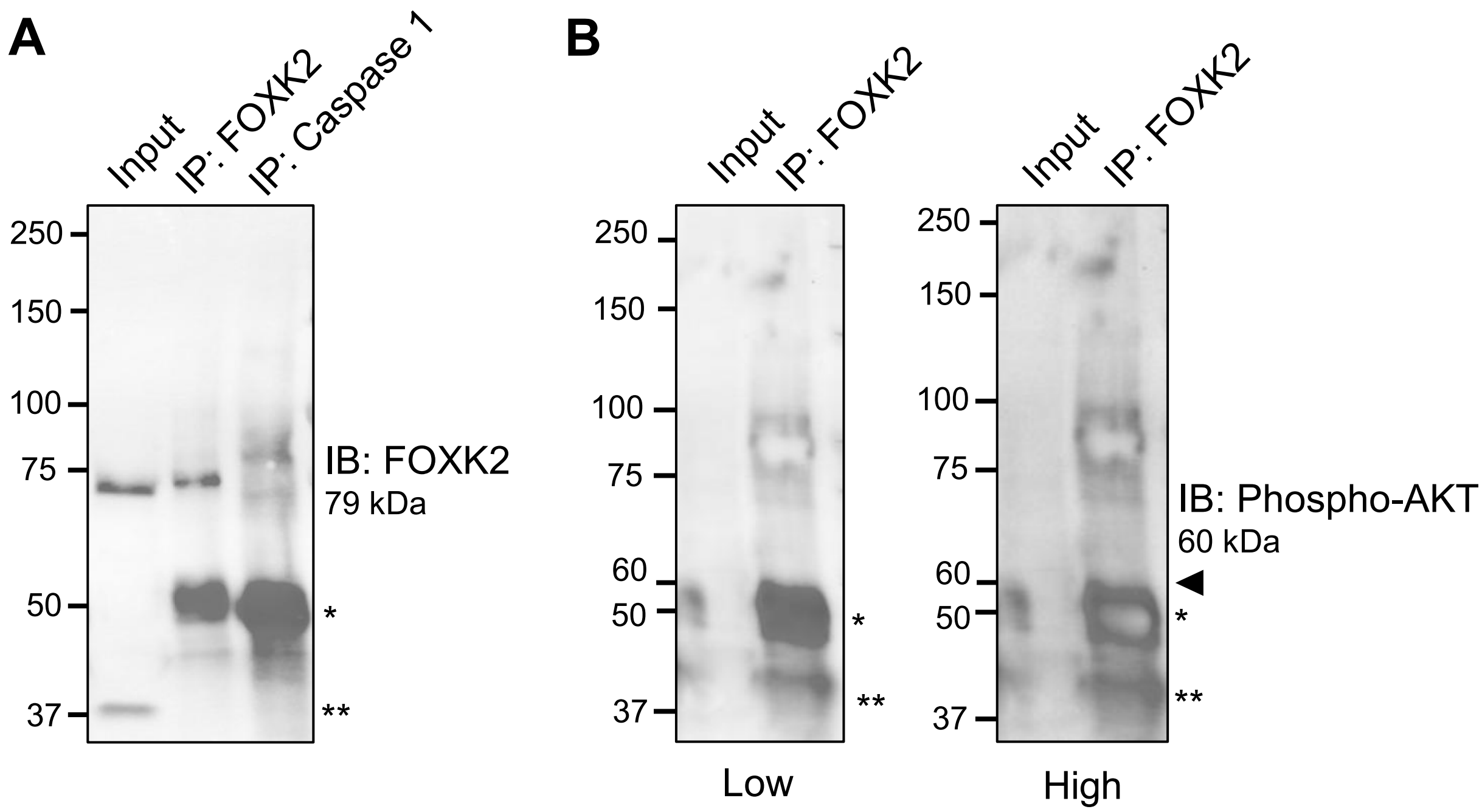


**Supplementary Fig. 14 – Phosphorylated AKT staining following FOXK2 immunoprecipitation in MDA-MB-231 cells**. (**A** and **B**) MDA-MB-231 cells were subjected to immunoprecipitation (IP) with anti-FOXK2 antibody conjugated to A/G plus agarose resins. The immunoprecipitates were analyzed by immunoblotting (IB) using FOXK2 (A) and Phospho-AKT (B) antibodies. The resins conjugated to anti-caspase1 antibody were used as a negative control and the inputs were reserved after the pre-clear step. Single asterisks indicate 50 kDa bands that correspond to the heavy chains of the immunoglobulins used in the protocol. Double asterisks indicate non-specific bands.
